# Macrocyclic Pyridyl Polyoxazoles: Structure-Activity Studies of the Aminoalkyl Side-Chain on G-Quadruplex Stabilization and Cytotoxic Activity

**DOI:** 10.3390/molecules181011938

**Published:** 2013-09-26

**Authors:** Gifty Blankson, Suzanne G. Rzuczek, Cody Bishop, Daniel S. Pilch, Angela Liu, Leroy Liu, Edmond J. LaVoie, Joseph E. Rice

**Affiliations:** 1 Department of Medicinal Chemistry, Ernest Mario School of Pharmacy, Rutgers–The State University of New Jersey, 160 Frelinghuysen Road, Piscataway, NJ 08854, USA; 2 Department of Pharmacology, Rutgers Robert Wood Johnson Medical School, Piscataway, NJ 08854, USA; 3 The Cancer Institute of New Jersey, Rutgers–The State University of New Jersey, New Brunswick, NJ 08901, USA

**Keywords:** synthesis, macrocycle, G-quadruplex, G-quadruplex stabilizer, G-quadruplex ligands

## Abstract

Pyridyl polyoxazoles are 24-membered macrocyclic lactams comprised of a pyridine, four oxazoles and a phenyl ring. A derivative having a 2-(dimethylamino)ethyl chain attached to the 5-position of the phenyl ring was recently identified as a selective G-quadruplex stabilizer with excellent cytotoxic activity, and good *in vivo* anticancer activity against a human breast cancer xenograft in mice. Here we detail the synthesis of eight new dimethylamino-substituted pyridyl polyoxazoles in which the point of attachment to the macrocycle, as well as the distance between the amine and the macrocycle are varied. Each compound was evaluated for selective G-quadruplex stabilization and cytotoxic activity. The more active analogs have the amine either directly attached to, or separated from the phenyl ring by two methylene groups. There is a correlation between those macrocycles that are effective ligands for the stabilization of G-quadruplex DNA (ΔT_tran_ 15.5–24.6 °C) and cytotoxicity as observed in the human tumor cell lines, RPMI 8402 (IC_50_ 0.06–0.50 μM) and KB3-1 (IC_50_ 0.03–0.07 μM). These are highly selective G-quadruplex stabilizers, which should prove especially useful for evaluating both *in vitro* and *in vivo* mechanism(s) of biological activity associated with G-quaqdruplex ligands.

## 1. Introduction

Regions of DNA and RNA that are rich in guanosine are known to fold into G-quadruplexes [1]. A G-quadruplex is formed when several G-tetrads (square-planar arrays of four guanines (G-tetrads) held together by hydrogen bonds) are stacked one upon another. Such arrangements are stabilized by π-stacking interactions between the purines as well as by monovalent metal cations (usually K^+^ or Na^+^) sandwiched between the G-tetrads [2]. G-Quadruplexes have been identified *in vitro* in telomeres, in the promoter regions of several oncogenes, and in mRNA [3,4,5,6,7,8,9,10]. The identification of several helicases and resolvases from nuclei, that efficiently unwind G-quadruplex DNA, lends support to the idea that the formation and resolution of G-quadruplexes *in vivo* play a vital role in subcellular processes [3,11]. It has been suggested that G-quadruplexes might play a role in a number of human diseases [12]. As a result considerable effort has already been expended on the development of potential therapeutic agents that function by targeting G-quadruplex formation [13,14].

**Figure 1:**
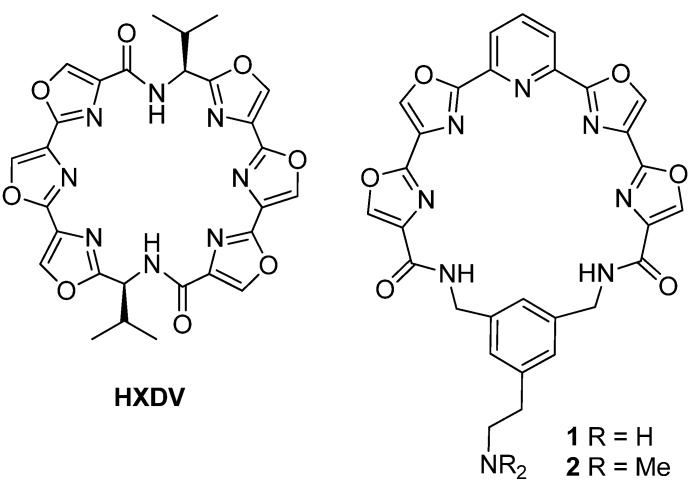
Structures of HXDV and pyridyl polyoxazole (PyPX) macrocycles **1** and **2**.

The development of G-quadruplex stabilizers as a potential new class of anticancer agents hinges on the ability to design compounds that stabilize only G-quadruplexes and not other nucleic acid structures such as duplex or triplex DNA [15]. While a diverse array of compounds have been reported to stabilize G-quadruplex DNA [1], most also have some ability to stabilize duplex DNA. The natural product telomestatin for example, is reported to stabilize G-quadruplex DNA with 70:1 selectivity over duplex DNA [16,17]. In contrast, the synthetic macrocyclic hexaoxazole HXDV (Figure 1) demonstrates no affinity for stabilizing single-stranded, duplex, or triplex DNA while strongly stabilizing G-quadruplex DNA [18,19]. HXDV induces apoptosis in both telomerase positive and negative cells, induces M-phase cell cycle arrest, reduces the expression of the M-phase checkpoint regulator Aurora A, and is moderately cytotoxic towards several tumor cell lines with an average IC_50_ value of 0.5 μM [18,20]. Unfortunately, the physicochemical properties of HXDV render it a poor candidate for *in vivo* evaluation. An extensive search for related compounds that retain exquisite selectivity for G-quadruplexes while displaying enhanced cytotoxic activity with improved solubility profiles led to the design and synthesis of a series of 24-membered macrocyclic pyridyl polyoxazoles (PyPX) [21]. Within this series compounds having a 1,3-bis(aminomethyl)phenyl group linking the ends of a pyridyl tetraoxazole dicarboxylate array were observed to be most cytotoxic when a 5-(2-aminoethyl)- (**1**, Figure 1) or a 5-(2-dimethylaminoethyl)- (**2**, Figure 1**)** substituent was attached to the phenyl ring. These analogs had IC_50_ values of 30-40 nM when assayed against KB3-1 cells and 90–180 nM against RPMI 8402 cells and strongly stabilize G-quadruplex DNA with no observable stabilization of duplex DNA. Compound **2** was selected for *in vivo* evaluation against a human breast cancer xenograft (MDA-MB-435) in athymic nude mice. Results from this assay indicated that mice treated with the pyridyl polyoxazole macrocycle had a %T/C value (average tumor volumes of treated/control animals) of 27.7% which clearly demonstrated *in vivo* efficacy against this breast cancer xenograft.

The initial structure-activity investigation as reported for the pyridy polyoxazole macrocycles suggests that a basic side-chain on the phenyl ring enhances cytotoxic activity and greatly improves the water-solubility of the macrocycle, allowing for easier formulation for *in vivo* evaluation [21]. In that report a 2-(*N,N*-dimethylamino)ethyl group was selected as the basic side-chain based on SAR obtained from the HX series of compounds [22]. The 5-position of the phenyl ring was chosen as the site of attachment based on the ease of synthesis. Herein we report the synthesis of analogs that have the side-chain attached to either the 4- or 5-position of the phenyl ring and that vary with respect to the number of spacer methylene groups connecting the tertiary amine to the ring. In addition, the preparation of analogs in which either one or two 2-(*N,N*-dimethylamino)ethyl groups are attached to the oxazole(s) that are more distant from the pyridine ring is also described. The effect of each of these structural changes on G-quadruplex selectivity and stabilization as well as on cytotoxic activity has been evaluated and is reported below.

## 2. Results and Discussion

### 2.1. Synthesis of Linkers Having a Basic Side-Chain at the 4-Position

**Scheme 1:**
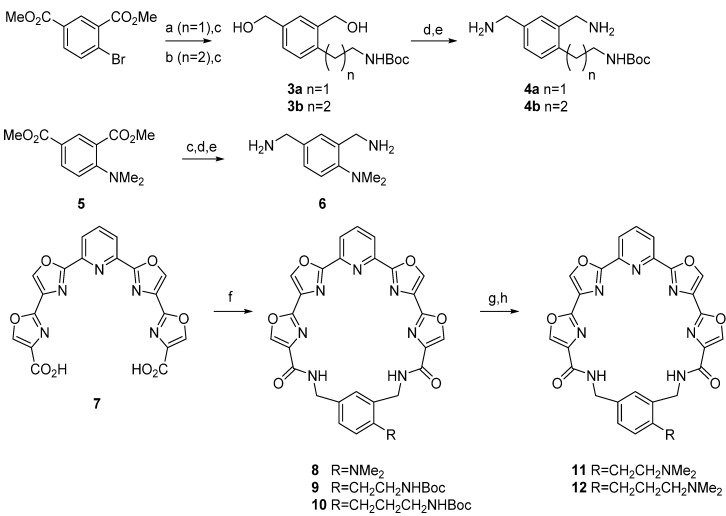
Synthesis of macrocycles having a basic side-chain at the 4-position.

The synthesis of diamine linkers having side-chains emanating from the 4-position of the phenyl ring is shown in Scheme 1. Those analogs having the *N,N*-dimethylamino group separated from the phenyl ring by two or three aliphatic carbons were prepared starting from dimethyl 4-bromoisophthalate [23]. The 2-aminoethyl analog was prepared by Suzuki reaction with potassium 2-[(*tert*-butoxycarbonylamino)ethyl]trifluoroborate [24] followed by lithium borohydride reduction to give diol **3a** in 56% yield. For the 3-aminopropyl analog hydroboration of N-Boc allylamine with 9-borobicyclo[3.3.1]nonane (9-BBN) followed by Suzuki coupling [25,26] of the derived borane with dimethyl 4-bromoisophthalate gave, after LiBH_4_ reduction, the three-carbon analog **3b** in 71% yield. Both diols were converted to their diazides with diphenyl phosphorylazide (DPPA), followed by reduction of the azide groups with polymer-supported triphenylphosphine to afford the bis(aminomethyl) derivatives **4a** and **4b** in good overall yield. The synthesis of a 4-(*N,N-*dimethylamino)methyl analog however proved challenging and despite much effort was not successful. An analog having a *N,N*-dimethylamino group directly attached to the phenyl ring at the 4-position was prepared started from the known dimethyl 4-(*N,N*-dimethylamino)isophthalate **5** [27]. The ester groups were reduced and the diol was converted into 1,3-bis(aminomethyl) derivative **6** as described above. Macrocyclization of the 4-substituted linkers was achieved by condensation of pentacyclic diacid **7** [21] in the presence of EDC and HOBt. The bis(lactams) **8**–**10** were prepared in yields ranging from 17%–18%. The N-Boc protected 2-aminoethyl and 3-aminopropyl compounds were deprotected using HCl and then converted to the *N,N*-dimethylamines **11** and **12** by reductive amination using formaldehyde and sodium triacetoxyborohydride.

*Reagents and conditions*: (**a**) KF_3_BCH_2_CH_2_NHBoc, PdCl_2_(dppf)CH_2_Cl_2_, 68%; (**b**) H_2_C=CHCH_2_NHBoc, 9-BBN, THF then PdCl_2_(dppf)CH_2_Cl_2_, Ph_3_As, CsCO_3_, DMF, 71%; (**c**) LiBH_4_, THF/EtOH, 87%–96%; (**d**) DPPA, DBU, THF, 44%–65%; (**e**) PS-PPh_3_, THF/H_2_O, 72%–95%; (**f**) **4a**, **4b**, or **6**, EDC, HOBt, DMF; (**g**) 20% HCl, MeOH/CH_2_Cl_2_, 100%; (**h**) HCHO (aq), NaBH(OAc)_3_, 23%–61%.

### 2.2. Synthesis of Linkers Having a Basic Side-Chain at the 5-Position

**Scheme 2:**
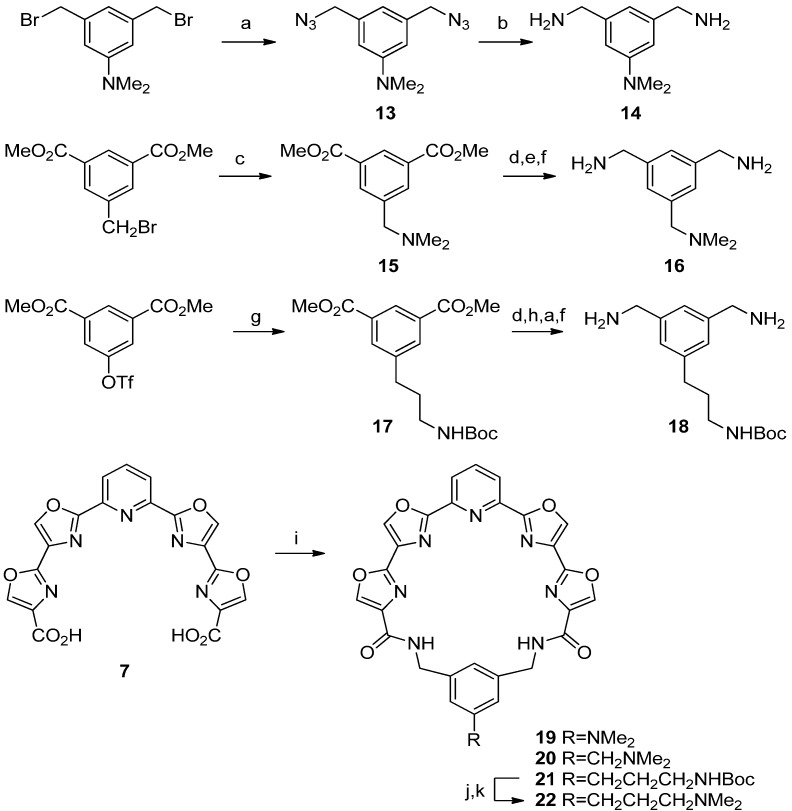
Synthesis of macrocycles having a basic side-chain at the 5-position.

Scheme 2 depicts the synthesis of macrocycles having a basic side-chain attached to the 5-position of the phenyl ring. For the synthesis of a linker molecule having a tertiary amine directly attached to the phenyl ring *N,N*-dimethyl 3,5-bis(bromomethyl)aniline [28] was treated with sodium azide to give a bis(azidomethyl) derivative **13** that was then reduced to diamine **14** using triphenylphosphine in aqueous THF. Synthesis of the 5-(*N,N*-dimethylaminomethyl) analog began by displacement of dimethyl 5-bromomethylisophthalate [29] by dimethylamine to afford **15** in 97% yield. The ester groups were then reduced with LiBH_4_, converted into the diazide derivative and reduced with triphenylphosphine to give the 1,3-bis(aminomethyl) derivative **16**.

*Reagents and conditions*: (**a**) NaN_3_, DMF, 90 °C, 73%; (**b**) PPh_3_, THF/H_2_O, 53%; (**c**) Me_2_NH, THF, 97%; (**d**) LiBH_4_, THF/EtOH, 62%; (**e**) DPPA, DBU, THF, 33%; (**f**) PS-PPh_3_, THF/H_2_O, 90%; (**g**) KF_3_BCH_2_CH_2_CH_2_NHBoc, RuPhos, Pd(OAc)_2_, Cs_2_CO_3_, THF, 95 °C, 71%; (**h**) MsCl, Et_3_N, CH_2_Cl_2_, 25%; (**i**) **14**, **16**, or **18**, EDC, HOBt, DMF; (**j**) TFA, CH_2_Cl_2_, 93%; (**k**) HCHO (aq), NaBH(OAc)_3_, MeOH/H_2_O, 48%.

Analog **2** possessing a 5-[2-(*N,N*-dimethylamino)ethyl] side chain has been synthesized previously [21]. Preparation of the 3-aminopropyl analog began with synthesis of potassium [3-(*tert*-butoxycarbonyl)amino)propyl]trifluoroborate, which was prepared in quantitative yield from N-Boc allylamine using the procedure detailed by Molander for the ethyl derivatives [24]. This was coupled with dimethyl 5-[(trifluoromethanesulfonyl)oxy]isophthalate [30] in the presence of palladium acetate, RuPhos, and cesium carbonate to give the N-Boc 3-aminopropyl derivative **17** in 71% yield. The conversion of **17** into diamine **18** was achieved by first reducing the ester groups to alcohols. The subsequent reaction with DPPA was not clean and therefore the diol was converted instead into a dimesylate derivative. Displacement with sodium azide afforded the diazide that was then reduced to give **18**. Macrocyclization of **14**, **16**, and **18** was performed by condensation with pentacyclic diacid **7** in the presence of EDC and HOBt to afford bis(lactams) **19**, **20**, and **21** in yields of 10%, 4%, and 43%. Compound **21** was treated with TFA to remove the Boc protecting group and the resulting amine was subjected to reductive amination as described above to give analog **22**.

### 2.3. Synthesis of Macrocycles Having the Basic Side-Chain(s) Located on Oxazole(s)

**Scheme 3:**
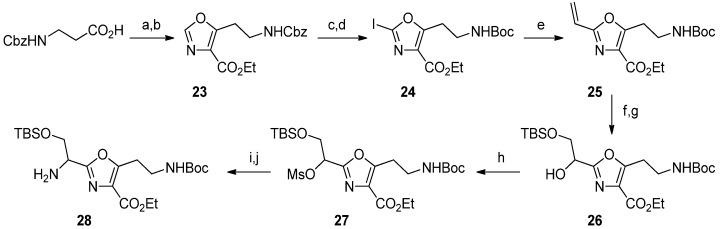
Synthesis of an oxazole intermediate having an aminoethyl side chain.

The synthesis of PyPX analogs having either a single or two 2-(*N,N*-dimethylamino)ethyl side chains on the oxazoles closer to the phenyl linker required the preparation of a suitable 5-substituted oxazole building block. This is detailed in Scheme 3 shown below. Starting from N-Cbz-β-alanine, treatment with oxalyl chloride gave the acid chloride which was reacted with ethyl isocyanoacetate in the presence of DBU to give oxazole **23** [31]. At this point a change in amine protecting group was deemed prudent, due to the difficulty in removing Cbz groups from intact macrocycles that we have observed in the PyPX series of compounds. Hydrogenolysis of the Cbz group in the presence of di-*tert*-butyl dicarbonate afforded the corresponding Boc-protected amine in high yield. Deprotonation of the remaining oxazole proton with LiHMDS, transmetallation to the zinc derivative and treatment with iodine occurred in one pot to give 2-iodooxazole **24** in 95% yield. Stille coupling with tributyl(vinyl)tin yielded the 2-vinyloxazole **25**, which was dihydroxylated using AD-mix-β [32]. The stereochemistry of the secondary alcohol is irrelevant since this stereocenter eventually becomes part of an oxazole ring, but the AD-mix procedure was more convenient and higher yielding than simple treatment with OsO_4_. The primary alcohol was selectively protected as TBS ether **26** and the remaining alcohol was converted into mesylate **27** in quantitative yield. Displacement of the mesylate with azide and reduction with polymer-supported triphenylphosphine completed the synthesis of alkylaminooxazole **28**.

*Reagents and conditions*: (**a**) (COCl)_2_; (**b**) CNCH_2_CO_2_Et, DBU, 65% (2 steps); (**c**) H_2_, Pd/C, Boc_2_O, 88%; (**d**) i. LiHMDS, THF, −42 °C; ii. ZnCl_2_, 0 °C; iii. I_2_, rt, 95%; (**e**) Bu_3_SnCH=CH_2_, Pd(PPh_3_)_2_Cl_2_, dioxane, 105 °C, 79%; (**f**) AD-mix-β, CH_3_SO_2_NH_2_, *t*-BuOH/H_2_O, rt, 61%; (**g**) TBSCl, imidazole, DMF, 0 °C, 79%; (**h**) MsCl, Et_3_N, CH_2_Cl_2_, 0 °C, 100%; (**i**) NaN_3_, DMF, rt, 100%; (**j**) PS-PPh_3_, THF/H_2_O, rt, 66%.

**Scheme 4:**
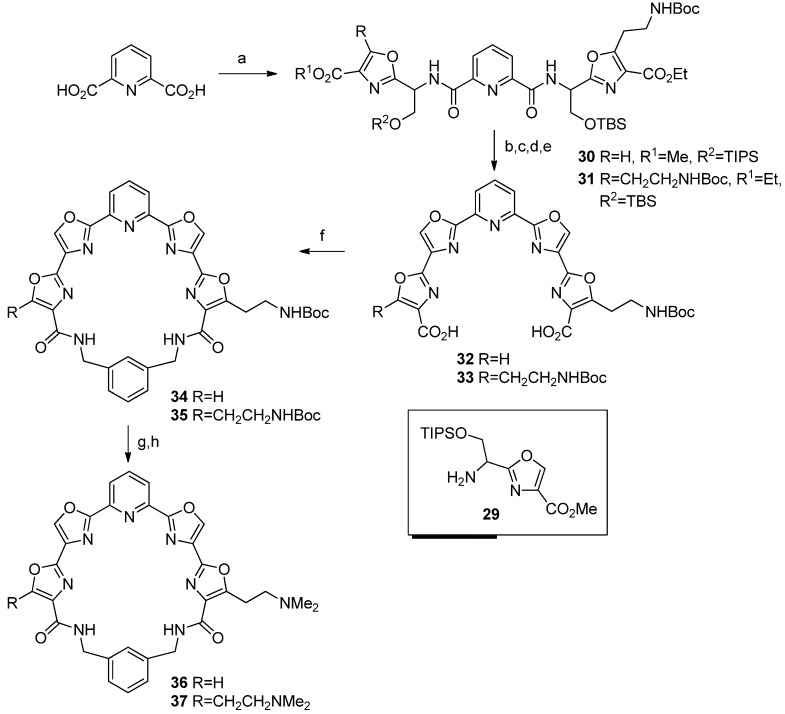
Synthesis of macrocycles having one or two aminoethyl side chains.

Elaboration of aminoalkyloxazole **28** into pyridyl tetraoxazole dicarboxylic acids having either one or two 2-(*N,N*-dimethylamino)ethyl side chains is outlined in Scheme 4. For the analog having a single aminoethyl side-chain pyridine-2,6-dicarboxylic acid was condensed with 0.6 equivalents of aminooxazole **29** [18] to limit the amount of diamide formed. The remaining carboxylic acid group was then condensed under the same conditions with 1 equivalent of aminooxazole **28** to give unsymmetrical diamide **30**. For the analog having two side-chains the pyridine dicarboxylic acid was condensed with 2 equivalents of oxazole **28** to give the symmetrical diamide **31**. In both cases the silyl ethers were removed by treatment with pyridine-HF complex and the resulting alcohols were treated with DAST and then BrCCl_3_ [33,34] to give the pyridyl tetraoxazoles **32** and **33** which were then hydrolyzed and macrocyclized with 1,3-bis(aminomethyl)benzene in the presence of MnSO_4_ to give **34** and **35**. We had found MnSO_4_ to sometimes have a beneficial templating effect on such macrocyclizations, although in this case the yields were in the 20%–28% range. The macrocycles were treated with TFA to remove the Boc protecting groups and the resulting amines were subjected to reductive amination to give the corresponding *N,N*-dimethyl amines **36** and **37**.

*Reagents and conditions*: (**a**) for **30**: EDC, HOBt, 2,6-lutidine, **29** (0.6 equiv.), 27%, then **28**, 66%; for **31:** EDC, HOBt, 2,6-lutidine, **28** (2 equiv), 85%; (**b**) HF-pyridine, THF, rt, 89%–100%; (**c**) DAST, CH_2_Cl_2_, −78 °C, then K_2_CO_3_, 91%–93%; (**d**) BrCCl_3_, DBU, CH_3_CN, 0 °C, 50%–60%; (**e**) LiOH, THF/H_2_O, 65%–67%; (**f**) 1,3-bis(aminomethyl)benzene, EDC, HOBt, 2,6-lutidine, MnSO_4_, DMF, rt, 2 d, 20%–28%; (**g**) TFA, CH_2_Cl_2_, 0 °C, 100%; (**h**) HCHO (aq), NaBH(OAc)_3_, MeOH/CH_2_Cl_2_, 100%.

### 2.4. Evaluation of G-Quadruplex Stabilization and Selectivity

Each compound was evaluated for its ability to selectively bind and stabilize G-quadruplex DNA in the presence of K^+^ ions (150 mM). Salmon testes (ST) DNA was employed as a model of duplex DNA and the human telomeric sequence d[T_2_G_3_(T_2_AG_3_)_3_A], denoted as hTel, was used as a model of quadruplex DNA. This sequence has been shown by Patel and co-workers, to exist as an intramolecular (3 + 1) G-quadruplex in which three strands are oriented in one direction and the fourth is oriented in the opposite direction in K^+^ solution [35]. The first-derivative forms of the UV melting profiles for ST DNA and hTel DNA were recorded in the absence and presence of the various macrocycles. The ligand-induced changes, if any, in the transition temperature (T*_tran_*) corresponding to the maxima (for quadruplex DNA) or minima (for duplex DNA) of these first-derivative melting profiles are listed in Table 1 for each macrocycle. With the exception of **8** which has a slight (<1 °C) destabilizing effect, none of the other macrocycles alters the thermal stability of ST duplex DNA to any significant extent and any observed changes in T*_tran_* are within the experimental uncertainty. This observation is consistent with little or no duplex DNA binding by these macrocycles. Similar behavior has been observed for other macrocyclic pyridyl polyoxazoles [21].

The results observed with the hTel quadruplex DNA however, stand in stark contrast with the ST duplex DNA results. In the series of three 4-phenyl substituted analogs compound **8** and **11** strongly stabilize G-quadruplex DNA by 20.5 and 24.6 °C respectively. Compound **8** has the tertiary amine directly connected to the phenyl while in **11** the amine is separated from the phenyl ring by two methylene groups. In contrast, the 3-dimethylaminopropyl analog **12** stabilizes G-quadruplex DNA to a much lesser extent. When the side-chain is attached to the 5-position of the phenyl ring the results are quite dramatic with the arylamine **19**, the previously-reported 2-(dimethylamino)ethyl analog **2** [21], and the propyl analog **22** all displaying strong stabilization of G-quadruplex DNA with ΔT_tran_ values of 15.5, 20.5 and 28.6 °C respectively. In striking contrast however is the dimethylaminomethyl analog **20** which has no significant stabilization (ΔT_tran_ = 0.1 °C) of G-quadruplex DNA. Quadruplex stabilization is also considerably less efficient when the side chain(s) are moved away from the phenyl ring and onto one or two of the oxazole rings. A 2-(dimethylamino)ethyl chain attached to one oxazole ring provides for weak G-quadruplex stabilization (ΔT_tran_ = 4.6 °C) while two such side-chains result in an even lower degree of stabilization (ΔT_tran_ = 1.6 °C). Most of these 4- and 5-phenyl substituted macrocyclic pyridyl polyoxazole analogs are stronger G-quadruplex stabilizers than HXDV (ΔT_tran_ = 11.5 °C) [21].

**Table 1:** Effect of various pyridyl polyoxazoles on the thermal stabilities of duplex and quadruplex DNA.

			hTel -Quadruplex DNA		Salmon Testes Duplex DNA	
Compound	# CH_2_ Spacer Units	Attach. Pos.	T_tran_ (°C)	ΔT_tran_ (°C)^a^	T_tran_ (°C)	ΔT_tran_ (°C) ^a^
**None**	na	na	64.6	--	86.6	--
**8**	0	4	85.1	20.5	85.7	−0.9
**11 **	2	4	89.2	24.6	86.2	−0.4
**12**	3	4	71.7	7.1	86.2	−0.4
**19**	0	5	80.1	15.5	86.1	−0.5
**20**	1	5	64.7	0.1	86.2	−0.4
**Compound**	# **CH_2_ Spacer Units**	**Attach. Pos.**	**T_tran_ (°C)**	**ΔT_tran_ (°C)**	**T_tran_ (°C)**	**ΔT_tran_ (°C)**
**2**	2	5	--	20.5 ^b^	--	0 ^b^
**22**	3	5	93.2	28.6	86.2	−0.4
**36**	2	oxazole	69.2	4.6	86.2	−0.4
**37**	2	oxazole (x2)	66.2	1.6	86.2	−0.4
**HXDV**	na	na	--	11.5 ^b^	--	0 ^b^

*Notes*: ^a^ ΔT*_tran_* reflects the change in transition temperature (T*_tran_*) of the target nucleic acid induced by the presence of the substrate. Values of T*_tran_* were determined from the maxima or minima of first-derivative UV melting profiles. The uncertainty in the ΔT*_tran_* values is ± 0.5 °C. ^b^ Values from ref. [21]. na = not applicable.

### 2.5. Evaluation of Cytotoxic Activity

Each *N,N*-dimethylamino-substituted macrocycle was also evaluated for cytotoxic activity against a human lymphoblastoma RPMI 8402 and a human epidermoid carcinoma KB3-1 cell line (Table 2). The results from these assays are informative about the relationship of structure to cytotoxic activity among the PyPX macrocyclic G-quadruplex stabilizers. Of greatest significance, especially from a synthetic viewpoint, is that for any given side-chain (**8** *vs.* **19** and **11** *vs.* **2**) attachment at the 5-position of the phenyl ring provides superior cytotoxic activity than attachment at the 4-position. An explanation for this observation is not clear at this time. The three-carbon analogs **12** and **22** have nearly equivalent activity at both positions, but these longer-chain analogs are generally lower in activity than the two-carbon analogs. Attachment of the dimethylamino group directly to the phenyl ring (**8** and **19**) leads to compounds having the greatest cytotoxic potency at their respective points of attachment. Unfortunately, these compounds are significantly less water-soluble than those analogs in which the amine is separated from the phenyl ring by one or more methylene groups. In general analogs that have an even number (0 or 2) of methylene spacer groups are more cytotoxic than those with an odd number. The one-carbon analog at the 5-position (**20)**, displays especially poor cytotoxic potency. Moving the side-chain away from the phenyl ring and onto an oxazole ring also fails to improve cytotoxic activity. As we have noted previously, a compound having a single water-solubilizing 2-(dimethylamino)ethyl chain **36** is superior to an analog having two such substituents **37** [22,36]. While **36** has reasonable cytotoxic potency and good water-solubility, the synthesis of this analog was considerably more involved than compound **2**.

The results from these assays suggest that for the macrocyclic pyridyl polyoxazoles those compounds that are inefficient at stabilizing G-quadruplex DNA are in general the least cytotoxic against KB3-1 and RPMI 8402 cells. These results are consistent with those recently reported for a series of 24-membered oxazole-containing macrocycles with a biphenyl scaffold [37]. With the exception of **22** those analogs that strongly stabilize G-quadruplex DNA exhibit cytotoxic activity against KB3-1 cells with IC_50_ values ≤ 70 nM while their activity against RPMI 8402 is also good, but more variable. An earlier investigation of the effect of side-chain length on quadruplex stabilization and cytotoxic activity among hexaoxazole (HX) analogs showed a similar pattern of results. While the propyl (n = 3) analog was a slightly more efficient G-quadruplex stabilizer than the ethyl analog, the 2-(dimethylamino)ethyl analog was more cytotoxic. A lysinyl (n = 4) analog was neither stabilizing towards G-quadruplexes not cytotoxic (IC_50_ > 3 μM) [22]. The aryl amine analogs **8** and **19** and the 2-(dimethylamino)ethyl analogs **11** and **2** are strongly cytotoxic with excellent G-quadruplex stabilization. The lower water-solubility of the aryl amines however, makes them less suitable than the 2-(dimethylamino)ethyl analogs for possible *in vivo* evaluation.

**Table 2:** Relative cytotoxic activities. IC_50_ values (μM) ^a^.

Compound	# CH_2_ Spacer Units	Attach. Pos.	RPMI 8402	KB3-1 wt.
**8**	0	4	0.12 ^b^	0.03 ± 0.00
**11 **	2	4	0.5 ± 0.4	0.07 ± 0.07
**12**	3	4	0.4 ± 0.0	0.25 ± 0.07
**19**	0	5	0.06 ± 0.01	0.03 ± 0.00
**20**	1	5	7.0 ± 4.2	5.5 ± 0.7
**2**	2	5	0.18 ^c^	0.04 ^c^
**22 **	3	5	0.5 ± 0.1	0.22 ± 0.13
**36**	2	oxazole	0.28 ± 0.04	0.14 ± 0.02
**37**	2	oxazole (x2)	1.5 ± 0.0	0.4 ± 0.1
HXDV	na	na	0.54 ± 0.12	0.35 ± 0.08

*Notes*: ^a^ Values are the means of at least two determinations ± standard deviation; ^b^ Single determination; ^c^ Values from ref. [21]; na = not applicable.

## 3. Experimental

### 3.1. General

All reactions were conducted under an atmosphere of dry nitrogen in oven-dried glassware unless otherwise noted. THF was dried by distillation from sodium-benzophenone. Toluene, CH_2_Cl_2_, 2,6-lutidine, Et_3_N, pyridine, DBU, and CH_3_CN were freshly distilled from CaH_2_. Anhydrous DMF was obtained by stirring overnight over anhydrous CuSO_4_ followed by distillation under reduced pressure. All starting materials and reagents were commercially available and were used as received with the exception of **5**, **7**, and **29** which were prepared as described previously [18,21,27]. Flash chromatography was conducted using 230–400 mesh silica gel obtained from Dynamic Adsorbents, Inc. Melting points were obtained on a Thomas-Hoover apparatus and are uncorrected. Proton (400 MHz) and carbon (125 MHz) NMR spectra were recorded on a Bruker Avance III spectrometer in CDCl_3_ unless otherwise noted. Chemical shifts are reported as δ units relative to internal tetramethylsilane. IR spectra were recorded on a Thermo-Nicolet Avatar 360 FT instrument as thin films on NaCl unless otherwise noted. High resolution mass spectra were provided by the Washington University Mass Spectrometry Resource, St. Louis, MO.

*1,3-Bis(hydroxymethyl)-4-[2-[(tert-butoxycarbonyl)amino]ethyl]benzene* (**3a**). *Step A*. A mixture of dimethyl 4-bromoisophthalate (273 mg, 1 mmol), potassium 2-(*tert*-butoxycarbonylamino)ethyl trifluoroborate (301 mg, 1.2 mmol), Cs_2_CO_3_ (1.08 g, 3.3 mmol), and PdCl_2_(dppf)**^.^**CH_2_Cl_2_ (49 mg, 0.06 mmol) in toluene 3 mL and water 1 mL was heated overnight at 80 °C under N_2_ in a sealed tube. The reaction mixture was then cooled to room temperature and saturated aqueous NH_4_Cl was added. The mixture was extracted with CH_2_Cl_2_ and the organic layer was dried over MgSO_4_ and then the solvent was evaporated under reduced pressure. The crude product was purified by flash chromatography eluting with 0%–20% ethyl acetate in hexane to give 229 mg of a colorless oil that proved to be an inseparable mixture of the desired product and 2,2',4,4'-tetra(carbomethoxy)biphenyl. This mixture was carried on to the next step. *Step B*. The mixture from above was dissolved in anhydrous THF (10 mL) and cooled to 0 °C under N_2_ and treated with LiBH_4_ (80 mg, 6 mmol) followed by EtOH (1 mL). The reaction was allowed to warm to room temperature. After 24 h the reaction mixture was poured into water and extracted with EtOAc. The organic layer was washed with brine, dried over Na_2_SO_4_ and concentrated. Purification was effected by flash chromatography eluting with 0%–5% MeOH/CH_2_Cl_2_ to give **3a** as a colorless oil; 157 mg, 56% (two steps); ^1^H-NMR δ 7.14 (s, 1H), 7.02 (d, 1H, *J* = 8), 6.97 (d, 1H, *J* = 8), 5.18 (br s, 1H), 4.43 (s, 2H), 4.36 (s, 2H), 3.11 (m, 2H), 2.66 (t, 2H, *J =* 8), 1.30 (s, 9H); ^13^C-NMR δ 156.5, 139.4, 139.2, 136.2, 129.9, 127.5, 126.5, 79.4, 64.3, 62.4, 41.6, 32.4, 28.2.

*1,3-Bis(hydroxymethyl)-4-[3-[(tert-butoxycarbonyl)amino]propyl]benzene* (**3b**). *Step A*. A solution of N-Boc allylamine (432 mg, 2.75 mmol) in THF (5 mL) was flushed with nitrogen, treated with 9-BBN (358 mg, 2.75 mmol) and stirred at room temperature for 2 h. Degassed water (0.14 mL) was added and the mixture was then added *via* cannula to a flask containing dimethyl 4-bromoisophthalate (500 mg, 1.83 mmol), PdCl_2_(dppf)**^.^**CH_2_Cl_2_ (75 mg, 0.09 mmol), Ph_3_As (28 mg, 0.09 mmol), Cs_2_CO_3_ (1.79 g, 5.49 mmol) and DMF (5 mL) under nitrogen. The reaction was stirred at 60 °C overnight and then the solvents were removed under reduced pressure. The residue was poured into brine and extracted several times with ether. The organic layers were combined, washed with brine, and dried over Na_2_SO_4_, filtered, and evaporated. The residue was purified by flash chromatography eluting with 0%–50% EtOAc/hexanes to give a yellow oil; 457 mg, 71%; ^1^H-NMR δ 8.49 (s, 1H), 8.01 (d, 1H, *J =* 8), 7.30 (d, 1H, *J =* 8), 4.90 (s, 1H), 3.86 (s, 6H), 3.13 (m, 2H), 2.99 (t, 2H, *J =* 8), 1.77 (m, 2H), 1.40 (s, 9H); ^13^C-NMR δ 166.9, 166.1, 156.0, 148.9, 132.7, 132.6, 131.1, 129.5, 128.1, 78.9, 52.1, 52.0, 40.2, 31.6, 31.5, 28.4; *Step B*. Prepared using the procedure detailed above for **3a**, *StepB*. Purification was effected by flash chromatography eluting with 0%–5% MeOH/CH_2_Cl_2_ to obtain **3b** as a colorless oil; 259 mg, 88%; ^1^H-NMR δ 7.26 (s, 1H), 7.11 (d, 1H, *J* = 8), 7.07 (d, 1H, *J* = 8), 5.07 (br s, 1H), 4.52 (s, 2H), 4.47 (s, 2H), ), 4.12 (br s, 2H), 3.05 (t, 2H, *J =* 4), 2.57, (t, 2H, *J =* 8), 1.67(m, 2H), 1.42 (s, 9H); ^13^C-NMR δ 156.4, 138.9, 138.7, 138.6, 129.1, 127.1, 126.4, 79.2, 64.4, 62.2, 40.2, 31.1, 28.9, 28.4.

*1,3-Bis(aminomethyl)-4-[2-[(tert-butoxycarbonyl)amino]ethyl]benzene* (**4a**). *Step A*. A solution of **3a** (150 mg, 0.56 mmol) in THF (7.5 mL) was cooled to 0 °C under N_2_ and treated dropwise with diphenyl phosphorylazide (DPPA) (0.36 mL, 1.68 mmol) followed by 1,8-diazabicyclo[5.4.0]undec-7-ene (DBU) (0.25 mL, 1.68 mmol). Stirring continued at 0 °C for 4 h and the reaction was then allowed to warm overnight to room temperature. The solution was poured into 5% HCl and extracted with EtOAc. The organic layer was separated, washed with brine, dried over Na_2_SO_4_, filtered, and evaporated to a brown oil. Purification by flash chromatography eluting with 5%–10% EtOAc/hexane afforded the diazide as a colorless oil; 112 mg, 62%; ^1^H-NMR δ 7.24 (m, 3H), 4.60 (br s, 1H), 4.35 (s, 2H), 4.27 (s, 2H), 3.29 (m, 2H), 2.80 (t, 2H, *J* = 8), 1.37 (s, 9); ^13^C-NMR δ 155.8, 137.1, 134.3, 134.2, 130.8, 129.6, 128.5, 79.4, 54.3, 52.4, 41.4, 32.7, 28.4. *Step B*. The diazide (90 mg, 0.27 mmol) was dissolved in a mixture of THF and water (5:2) and polymer-supported triphenylphosphine (PS-PPh_3_) (227 mg, 3 mmol/g) was added. The mixture was stirred at room temperature overnight and then filtered and concentrated. The crude product was re-dissolved in toluene and evaporated several times to give **4a** as a yellow oil; 54 mg, 71%; ^1^H-NMR δ 7.10 (m, 3H), 5.56 (br s, 1H), 3.83 (s, 2H), 3.77 (s, 2H), 3.30 (m, 2H), 2.78 (t, 2H, *J =* 8), 1.35 (s, 9H); ^13^C-NMR δ 156.1, 141.7, 135.9, 130.1, 127.4, 126.1, 125.5, 78.9, 46.2, 43.7, 31.9, 30.3, 28.4.

*1,3-Bis(aminomethyl)-4-[3-[(tert-butoxycarbonyl)amino]propyl]benzene* (**4b**). *Step A*. Prepared using the procedure detailed above for **4a**, *Step A*. Purification was achieved by flash chromatography eluting with 0%–15% EtOAc/hexanes. A colorless oil was obtained; 113 mg, 65%; ^1^H-NMR δ 7.17 (m, 3H), 4.58 (br s, 1H), 4.30 (s, 2H), 4.26 (s, 2H), 3.12 (m, 2H), 2.61 (t, 2H, *J =* 8), 1.72 (m, 2H), 1.38 (s, 9H); ^13^C-NMR δ 155.9, 140.4, 133.7, 133.6, 130.1, 129.5, 128.5, 79.2, 54.3, 52.5, 40.3, 31.2, 29.3, 28.4. *Step B*. Prepared using the procedure detailed above for **4a**, *Step B*. The filtrate was azeotroped with toluene to obtain **4b** as a yellow oil; 73 mg, 96%; ^1^H NMR (CD_3_OD) δ 7.05 (m, 3H), 3.72 (s, 2H), 3.66 (s, 2H), 2.97 (m, 2H), 2.54 (t, 2H, *J =* 8), 1.61 (m, 2H), 1.32 (s, 9H); ^13^C-NMR δ 158.5, 140.8, 140.7, 139.5, 130.6, 128.4, 127.4, 79.9, 46.2, 43.3, 31.0, 30.2, 28.9.

*N,N-Dimethyl-2,4-bis(aminomethyl)aniline* (**6**). *Step A*. Prepared from **5** [27] using the procedure detailed above for **3a**, *Step B*. Flash chromatography eluting with 0%–4% MeOH/CH_2_Cl_2_ gave the diol as a yellow oil; 1.18 g, 90%; ^1^H-NMR δ 7.17 (dd, 1H, *J =* 1,8), 7.12 (d, 1H, *J =* 1), 7.09 (d, 1H, *J =* 8), 4.70 (s, 2H), 4.51 (s, 2H), 2.65 (s, 2H); ^13^C NMR δ 150.6, 137.5, 135.0, 127.3, 126.9, 120.1, 64.5, 64.3, 44.8. *Step B*. Prepared using the procedure detailed above for **4a**, *StepA*. Flash chromatography eluting with 0-4% EtOAc/hexane gave the diazide as a colorless oil; 287 mg, 44%; ^1^H-NMR δ 7.32 (d, 1H, *J =* 2), 7.28 (dd, 1H, *J =* 2,7), 7.18 (d, 1H*, J =* 7), 4.52 (s, 2H), 4.34 (s, 2H), 2.73 (s, 6H); ^13^C-NMR δ 152.9, 130.6, 130.4, 129.9, 128.9, 120.1, 54.3, 50.6, 45.1. *Step* **C**. Prepared using the procedure detailed above for **4a**, *Step B*. The residue was re-dissolved in toluene and evaporated several times to give **6** as a yellow oil; 161 mg, 83%; ^1^H-NMR δ 7.18 (d, 1H, *J =* 1.6), 7.07 (dd, 1H, *J =* 1.6, 8), 6.99 (d, 1H, *J =* 8), 3.82 (s, 2H), 3.72 (s, 2H), 2.59 (s, 6H); ^13^C-NMR δ 150.1, 137.2, 136.2, 126.4, 125.2, 118.6, 44.9, 44.1, 42.1.

*Pyridyl tetraoxazole macrocycle with a 4-(N,N-dimethylamino) group on the phenyl ring* (**8**). Diacid **7** [21] (121 mg, 0.28 mmol) was suspended in DMF (120 mL) and MnSO_4_•H_2_O (95 mg, 0.56 mmol) was added. The solution was warmed to 65 °C under N_2_ for 20 min and then cooled back to room temperature. EDC (213 mg, 1.1 mmol), HOBt (150 mg, 1.1 mmol), and 2,6-lutidine (239 mg, 2.3 mmol) were added followed by the slow drop-wise addition of a solution of **9** (50 mg, 0.28 mmol) in DMF (5 mL). The solution was stirred at room temperature for 48 h and then the white precipitate was filtered off and purified by flash chromatography eluting with 0%–10% MeOH/CH_2_Cl_2_ to give macrocycle **8** as a white solid; 27 mg, 17%; mp 268–270 °C; ^1^H-NMR (CDCl_3_ + CD_3_OD) δ 8.21 (s, 1H), 8.19 (s, 2H), 8.16 (s, 1H), 7.99 (m, 3H), 7.62 (m, 1H), 7.27 (m, 3H), 7.07 (d, 1H, *J =* 8), 4.63 (d, 2H, *J =* 4), 4.47 (d, 2H, *J =* 4 ), 2.61 (s, 6H); ^13^C-NMR (CDCl_3_ + CD_3_OD) δ 160.5, 160.4, 160.1, 159.5, 154.3, 154.2, 153.7, 145.5, 145.4, 140.7, 140.4, 139.2, 138.8, 138.5, 137.5, 137.5, 132.5, 132.1, 131.94, 131.8, 131.1, 130.5, 122.7, 122.6, 121.1, 45.1, 42.9, 40.4; HRMS (ESI) *m/z* calcd for C_29_H_22_N_8_O_6_ (M+H): 579.1735; found: 579.1737.

*Pyridyl tetraoxazole macrocycle with a 4-[2-[(tert-butoxycarbonyl)amino]ethyl]group on the phenyl ring* (**9**). Prepared using the procedure detailed above for **8**. White solid; 18 mg, 18%; mp 198–200 °C; ^1^H-NMR δ 8.32 (s, 2H), 8.27 (s, 2H), 8.05 (m, 3H), 7.41 (s, 1H), 7.25 (d, 1H, *J =* 8), 7.22 (d, 1H, *J =* 8), 5.42 (br s, 1H), 4.59 (s, 2H), 4.49 (s, 2H), 3.17 (m, 2H), 2.78 (t, 2H, *J =* 8), 1.32 (s, 9H); ^13^C-NMR δ 160.7, 159.9, 154.3, 144.9, 141.5, 141.46, 139.5, 138.9, 138.0, 137.2, 135.3, 132.4, 131.5, 130.9, 130.85, 129.6, 122.8, 79.1, 43.6, 41.2, 38.7, 32.5, 28.9; HRMS (ESI) *m/z* calcd for C_34_H_30_N_8_O_8_ (M+Na): 701.2084; found: 701.2075.

*Pyridyl tetraoxazole macrocycle with a 4-[3-[(tert-butoxycarbonyl)amino]propyl]group on the phenyl ring* (**10**). Prepared using the procedure detailed above for **8**. Off-white solid; 21.2 mg, 18%; mp 190–192 °C; ^1^H-NMR δ 8.39 (d, 2H, *J =* 8), 8.20 (m, 4H), 8.01 (m, 3H), 7.30 (s, 1H), 7.23 (d, 1H, *J =* 8), 7.16 (d, 1H, *J =* 8), 4.81 (br s, 1H), 4.59 (s, 2H), 4.48 (s, 2H), 3.07(m, 2H), 2.61 (m, 2H), 1.68 (m, 2H), 1.36 (s, 9H); ^13^C-NMR δ 160.5, 159.7, 159.4, 156.0, 154.2, 145.3, 140.9, 140.7, 139.0, 138.4, 137.5, 137.2, 135.2, 132.0, 131.9, 131.8, 130.0, 122.8, 122.7, 79.1, 43.7, 41.5, 31.9, 29.6, 29.2, 28.4; HRMS (ESI) m/z C_35_H_32_N_8_O_8_ (M+H): 693.2425; found: 693.2416.

*Pyridyl tetraoxazole macrocycle with a 4-[2-(N,N-dimethylamino)ethyl] group on the phenyl ring* (**11**). *Step A*. N-Boc derivative **9** (7 mg, 0.0103 mmol) was suspended in 20% HCl (1 mL) and stirred at room temperature for 2 h. The solution was evaporated under reduced pressure to give the amine salt that was used directly for the next step. *Step B*. The salt from *Step A* (7 mg, 0.01 mmol) was suspended in 20% MeOH/CH_2_Cl_2_ (3 mL) and treated with 37% aqueous formaldehyde (0.5 mL). After stirring for 5 min at room temperature sodium triacetoxyborohydride (24 mg, 0.114 mmol) was added in one portion and stirring was continued overnight. The reaction mixture was partitioned between saturated NaHCO_3_ and CH_2_Cl_2_, and the organic extract was dried over Na_2_SO_4_, filtered and evaporated to a solid. Purification was performed by Chromatotron (SiO_2_, 1 mm rotor) eluting with 1%–20% MeOH/CH_2_Cl_2_ + 1% NH_4_OH to give compound **11** as a pale yellow solid; 1.5 mg, 23%; mp 278–280 °C (dec.); ^1^H-NMR δ 8.25 (m, 4H), 8.02 (m, 3H), 7.39 (s, 1H), 7.24 (d, 1H, *J =* 8), 7.19 (d, 1H, *J =* 8), 4.60 (s, 2H), 4.50 (s, 2H), 2.87 (t, 2H, *J =* 8), 2.57 (t, 2H, *J =* 8), 2.23(s, 6H); ^13^C-NMR δ 160.6, 159.9, 159.6, 154.2, 154.1, 145.1, 141.2, 139.2, 138.9, 138.7, 137.6, 137.5, 135.1, 132.4, 131.7, 130.7, 129.7, 122.7, 60.8, 44.7, 43.6, 41.32, 29.7; HRMS (ESI) *m/z* calcd for C_31_H_26_N_8_O_6_ (M+H): 607.2040; found: 607.2048.

*Pyridyl tetraoxazole macrocycle with a 4-[3-(N,N-dimethylamino)propyl] group on the phenyl ring* (**12**). *Step A*. Prepared from **10** using the procedure detailed above for **11**, *Step A*. The product from this reaction was taken directly to the next step without purification. *Step B*. Prepared using the procedure detailed above for **11**, *Step B*. Off-white solid; 4.8 mg, 61%; mp 202–205 °C; ^1^H-NMR δ 8.40 (d, 2H, *J =* 8), 8.22 (m, 4H), 8.01 (m, 3H), 7.33 (s, 1H), 7.25 (d, 1H, *J =* 8), 7.15 (d, 1H, *J =* 8), 4.59 (s, 2H), 4.49 (s, 2H), 2.60 (m, 2H) 2.31 (m, 2H), 2.20 (s, 6H), 1.7 (m, 2H); ^13^C-NMR δ 160.5, 159.6, 159.3, 154.2, 145.4, 140.6, 139.0, 138.4, 137.5, 137.3, 135.0, 131.9, 131.8, 130.7, 130.0, 122.7, 58.9, 44.8, 43.6, 41.5, 30.1, 29.3; HRMS (ESI) calculated for C_32_H_26_N_8_O_6_ (M+H): 621.2209; found: 621.2204.

*N,N-Dimethyl-3,5-bis(azidomethyl)aniline* (**13**). *N,N*-Dimethyl-3,5-bis(bromomethyl)aniline [28] (100 mg, 0.33 mmol) was dissolved in anhydrous DMF (10 mL) and sodium azide (128 mg, 1.97 mmol) was added. The reaction mixture was placed under argon and heated to 90 °C overnight. After cooling to room temperature, the reaction was poured into water and extracted with CH_2_Cl_2_. The combined organic extracts were dried with Na_2_SO_4_ and evaporated under reduced pressure to give diazide **13** as a pale orange oil; 71 mg, 73%; ^1^H-NMR δ 6.59 (s, 3H), 4.29 (s, 4H), 2.98 (s, 6H); ^13^C-NMR δ 151.2, 136.9, 115.7, 111.7, 55.2, 40.4; IR 3346, 2876, 2813, 2479, 2098, 1604, 1493, 1443, 1372, 1254, 1165, 1063, 1030, 986, 829, 724 cm^−1^.

*N,N-Dimethyl-3,5-bis(aminomethyl)aniline* (**14**). Prepared using the procedure detailed above for **4a**, *Step B*. The solvent was removed under reduced pressure to afford **14** as a colorless oil; 30 mg, 53%; ^1^H-NMR δ 6.57 (s, 3H), 3.81 (s, 4H), 2.96 (s, 6H); ^13^C-NMR δ 151.2, 144.7, 114.4, 110.2, 47.0, 40.7; IR 3355, 2914, 2360, 1601, 1488, 1442, 1361, 1317, 1230, 1165, 1132, 1061, 995, 834, 700 cm^−1^; HRMS (ESI) *m/z* calcd for C_10_H_18_N_3_ (M+H): 180.1501; found: 180.1502.

*Dimethyl 5-(N,N-Dimethyl)aminoisophthalate* (**15**). A solution of dimethyl 5-(bromomethyl)iso­phthalate [29] (890 mg, 3.11 mmol) in anhydrous THF (15 mL) was treated at room temperature with dimethylamine (10 mL, 20 mmol, 2M in THF). This was stirred for 30 min during which time a white solid precipitated. The mixture was poured into 1N NaOH and extracted with EtOAc. The organic layer were washed with brine and dried over Na_2_SO_4_. Concentration under reduced pressure gave **15** as a yellow oil; 761 mg, 97%; ^1^H-NMR δ 8.59 (s, 1H), 8.19 (s, 2H), 3.95 (s, 6H), 3.51 (s, 2H), 2.26 (s, 6H); ^13^C-NMR δ 166.3, 140.2, 134.4, 134.3, 130.7, 129.6, 128.8, 63.5, 52.3, 45.4; IR 3434, 2951, 2856, 2820, 2776, 2256, 1728, 1640, 1606, 1456, 1435, 1366, 1329, 1244, 1205, 1148, 1122, 1107, 1043, 1008, 913, 873, 842, 790, 755, 733, 647 cm^−1^; HRMS (ESI) *m/z* calcd for C_13_H_17_NO_4_ (M+H): 252.1230; found: 252.1237.

*1,3-Bis(aminomethyl)-5-(N,N-dimethylamino)methylbenzene* (**16**). *Step A*. Prepared using the procedure detailed above for **3a**, *Step B*. Concentration gave a yellow oil that was purified by flash chromatography eluting with 1%–20% MeOH/CH_2_Cl_2_. The diol was isolated as a white solid; 222 mg, 38%; mp 94–95 °C; ^1^H-NMR δ 7.42 (s, 1H), 7.27 (s, 2H), 4.75 (s, 4H), 3.99 (s, 2H), 2.53 (s, 6H); ^13^C-NMR δ 141.6, 132.0, 129.9, 126.0, 67.5, 64.8, 50.0; IR 3396, 3004, 2950, 2370, 2271, 1644, 1525, 1468, 1368, 1168, 1018, 873, 847, 821 cm^−1^; HRMS (ESI) *m/z* calcd for C_11_H_17_NO_2_ (M+H): 196.1332; found: 196.1331. *Step B*. Prepared using the procedure detailed above for **4a**, *Step A*. Purification by flash chromatography eluting with 10%–30% EtOAc/hexane afforded the diazide as a colorless oil; 200 mg, 72%; ^1^H-NMR δ 7.30 (m, 3H), 4.43 (s, 4H), 4.01 (s, 2H), 2.56 (s, 6H); ^13^C-NMR δ 136.6, 132.7, 131.7, 125.6, 67.1, 54.1, 50.1; IR 2950, 2372, 2272, 2097, 1693, 1464, 1345, 1246, 1169, 1017, 842, 819 cm^−1^; HRMS (ESI) *m/z* calcd for C_11_H_15_N_7_ (M+H): 246.1462; found: 246.1460. *Step C*. Prepared using the procedure detailed above for **4a**, *Step B*. **16** was obtained as a colorless oil; 77 mg, 100%; ^1^H-NMR δ 7.37 (s, 1H), 7.22 (s, 2H), 3.82 (m, 6H), 2.49 (s, 6H); ^13^C-NMR δ 143.6, 130.6, 129.8, 120.1, 67.4, 66.2, 49.7; IR 3314, 2945, 2369, 2318, 2271, 1666, 1605, 1467, 1169, 1017, 819 cm^−1^; HRMS (ESI) *m/z* calcd for C_11_H_19_N_3_ (M+H): 194.1652; found: 194.1649.

*Dimethyl 5-[3-[(tert-Butoxycarbonyl)amino]propyl]isophthalate* (**17**). A reusable sealed-tube was equipped with a magnetic stirrer and charged with dimethyl 5-(trifluoromethanesulfonyl­oxy)isophthalate [30] (342 mg, 1 mmol), potassium 3-(*tert*-butoxycarbonylamino)propyltrifluoroborate (265 mg, 1 mmol), RuPhos (46.7 mg, 0.1 mmol), Cs_2_CO_3_ (977 mg, 3 mmol), Pd(OAc)_2_ (11.2 mg, 0.05 mmol), toluene (3 mL), and water (1 mL) and was sparged with nitrogen for a few minutes and then tightly sealed. The tube was placed into a pre-heated oil bath at 95 °C for 23 h. The tube was allowed to come to room temperature, sat. NH_4_Cl (16 mL) was added, and the mixture was extracted with CH_2_Cl_2_ (3 × 20 mL). The combined extracts were dried over Na_2_SO_4_, filtered and evaporated under reduced pressure to give the crude product as a yellow oil. Purification by flash chromatography on silica gel eluting with 25% EtOAc/hexanes afforded **17** as a colorless oil; 250 mg, 71%; ^1^H-NMR δ 8.49 (s, 1H), 8.03 (s, 2H), 4.59 (br s, 1H), 3.92 (s, 6H), 3.14 (t, 2H, *J =* 7), 2.73 (t, 2H, *J =* 7), 1.83 (tt, 2H, *J =* 7,7), 1.42 (s, 9H); ^13^C-NMR δ 166.3, 155.9, 142.5, 133.7, 130.7, 128.5, 79.3, 52.3, 40.1, 32.7, 31.6, 28.4.

*1,3-Bis(aminomethyl)-5-[3-[(tert-butoxycarbonyl)amino]propyl]benzene* (**18**). *Step A*. Prepared using the procedure detailed above for **3a**, *Step B*. The diol was obtained as a colorless oil; 800 mg, 98%; ^1^H-NMR δ 7.09 (s, 1H), 7.02 (s, 2H), 4.75 (br s, 1H), 4.53 (s, 4H), 3.47 (br s, 2H), 3.04 (t, 2H, *J =* 7), 2.57 (t, 2H, *J* = 7), 1.73 (m, 2H), 1.42 (s, 9H); ^13^C-NMR δ 156.2, 141.9, 141.4, 126.1, 123.1, 79.4, 64.8, 40.2, 32.8, 31.4, 28.4. *Step B*. The diol (710 mg, 2.4 mmol) was dissolved in CH_2_Cl_2_ (24 mL), treated with Et_3_N (1.1 mL, 8 mmol), and cooled to 0 °C under a drying tube. Methanesulfonyl chloride (0.62 mL, 8 mmol) was added and the solution was stirred for 5.5 h and then poured into water (25 mL). The organic layer was separated and washed with water (10 mL) and then dried over Na_2_SO_4_, filtered and evaporated to give a pale-yellow oil. Flash chromatography on silica gel eluting with 2:1 EtOAc/hexanes afforded the dimesylate as a colorless oil; 270 mg, 25%; ^1^H-NMR δ 7.30 (s, 1H), 7.27 (s, 2H), 5.22 (s, 4H), 4.66 (br s, 1H), 3.14 (t, 2H, *J =* 7), 2.99 (s, 6H), 2.67 (t, 2H, *J =* 7), 1.82 (tt, 2H, *J =* 7,7), 1.45 (s, 9H); ^13^C-NMR δ 156.0, 143.4, 134.5, 129.5, 126.4, 79.3, 70.7, 40.0, 38.2, 32.7, 31.6, 28.4. *Step C*. Prepared using the procedure detailed above for **13**. The diazide was obtained as a colorless oil; 180 mg, 87%; ^1^H-NMR δ 7.11 (s, 3H), 4.56 (br s, 1H), 4.34 (s, 4H), 3.16 (t, 2H, *J =* 7), 2.68 (t, 2H, *J =* 7), 1.83 (m, 2H), 1.46 (s, 9H); ^13^C-NMR δ 156.0, 143.1, 136.3, 128.1, 125.6, 77.3, 54.6, 40.3, 32.9, 31.7, 28.4. *Step D*. Prepared using the procedure detailed above for **4a**, *Step B*. Diamine **18** is a pale-yellow oil; 140 mg, 90%; ^1^H NMR δ 7.09 (s, 1H), 7.00 (s, 2H), 4.58 (br s, 1H), 3.83 (s, 4H), 3.13 (m, 2H), 2.62 (t, 2H, *J =* 7), 1.80 (m, 6H (H2' + 2NH_2_)), 1.44 (s, 9H); ^13^C-NMR δ 156.0, 143.6, 142.2, 125.7, 123.6, 79.1, 46.4, 40.2, 33.0, 31.7, 28.4.

*Pyridyl tetraoxazole macrocycle with a 5-N,N-dimethylamino group on the phenyl ring* (**19**). Prepared using the procedure detailed above for **8**. White solid; 9 mg, 10%; mp 275–280 °C (dec.); ^1^H-NMR δ 8.32 (m, 6H), 8.10 (m, 3H), 6.90 (s, 1H), 6.71 (s, 2H), 4.56 (s, 4H), 2.93 (s, 6H); ^13^C-NMR δ 160.8, 160.3, 154.3, 151.7, 145.2, 141.4, 139.4, 138.9, 138.4, 137.7, 131.8, 122.8, 118.3, 113.3, 44.0, 40.6; IR 3417, 1644, 1605, 1439, 1370, 1172, 1112, 926 cm^−1^; HRMS (ESI) *m/z* calcd for C_29_H_23_N_8_O_6_ (M+H): 579.1735; found: 579.1727.

*Pyridyl tetraoxazole macrocycle with a 5-(N,N-dimethylaminomethyl) group on the phenyl ring* (**20**). Prepared using the procedure detailed above for **8**. White solid; 5 mg, 4%; mp > 300 °C; ^1^H-NMR δ 8.58 (s, 2H), 8.44 (m, 4H), 8.63 (s, 2H), 8.07 (m, 2H), 3.97 (m, 6H), 3.46 (s, 3H), 3.12 (s, 3H); HRMS (ESI) *m/z* calcd for C_30_H_24_N_8_O_6_ (M+H) 593.1892; found: 593.1885.

*Pyridyl tetraoxazole macrocycle with a 5-[3-[(tert-butoxycarbonyl)amino]propyl] group on the phenyl ring* (**21**). Prepared using the procedure detailed above for **8**. White solid; 40 mg, 43%; ^1^H-NMR δ 8.27 (s, 2H, H_5_ oxazole), 8.25 (s, 2H, H_5_ oxazole), 8.06 (m, 3H, H_3-5_ pyr), 7.52 (t, 2H, *J =* 5, lactam N*H*), 7.23 (s, 1H, H_2_ phenyl), 7.15 (s, 2H, H_4,6_ phenyl), 4.59 (br s, 1H, Boc N*H*), 4.55 (d, 4H, *J* = 5, C*H*_2_NHC=O), 3.10 (m, 2H, H_3'_), 2.60 (t, 2H, *J =* 7, H_1'_), 1.78 (m, 2H, H_2'_), 1.39 (s, 9H, *Me*_3_); ^13^C-NMR δ 160.5 (*C*=O lactam), 159.7 (C_2,6_ pyridine), 156.0 (C=O carbamate), 154.2 (C_2_ distal oxazole), 145.4 (C_2_ proximal oxazole), 143.6 (C_1,3_ phenyl), 142.7 (C_5_ phenyl), 140.6 (C_5_ prox. oxazole), 139.1 (C_5_ distal oxazole), 138.5 (C_4_ pyr), 137.7 (C_4_ distal oxazole), 131.8 (C_4_ (proximal oxazole), 129.5 (C_4,6_ phenyl), 127.8 (C_2_ phenyl), 122.7 (C_3,5_ pyr), 79.1 (*C*Me_3_), 43.8 (*C*H_2_NHC=O), 40.2 (C_3'_), 32.8 (C_1'_), 31.7 (C_2'_), 28.4 (Me_3_).

*Pyridyl tetraoxazole macrocycle with a 5-[3-(N,N-dimethylamino)propyl] group on the phenyl ring* (**22**). *Step A*. Prepared from **21** using the procedure detailed above for **11**, *Step A*, but substituting 1:1 TFA/CH_2_Cl_2_ for 20% HCl. The trifluoroacetate salt is a white solid; 38 mg, 93%. *Step B*. The salt was converted into **22** using the procedure detailed above for **11** *Step B*. White solid; 16 mg, 48%; ^1^H-NMR δ 8.28 (s, 2H, H_5_ distal oxazole), 8.19 (s, 2H, H_5_ proximal oxazole), 7.98 (s, 3H, H_3-5_ pyridine), 7.21(s, 1H, H_2_ phenyl), 7.03 (s, 2H, H_4,6_ phenyl), 4.42 (s, 4H, C*H*_2_ macrocycle), 2.49 (t, 2H, *J =* 7, H_1'_), 2.41 (t, 2H, *J =* 7, H_3'_), 2.24 (s, 6H, *Me*_2_N), 1.74 (m, 2H, H_2'_); ^13^C-NMR δ 160.6 (*C*=O), 160.2 (C_2,6_ pyr), 154.4 (C_2_ dist. oxaz.), 145.0 (C_2_ prox. oxaz.), 141.2 (C_5_ prox. oxaz.), 139.6 (C_5_ dist. oxaz.), 138.7 (C_4_ pyr), 137.7 (C_4_ dist. oxaz.), 131.3 (C_4_ prox. oxaz.), 129.0 (C_4,6_ phen), 127.6 (C_2_ phen), 122.8 (C_3,5_ pyr.), 58.4 (C_3'_), 43.9 (*Me*_2_N), 43.5 (*C*H_2_ macrocycle), 32.8 (C_1'_), 27.4 (C_2'_); HRMS (ESI) *m/z* calcd for C_32_H_28_N_8_O_6_ (M+H) 621.2210; found: 621.2205.

*Ethyl 5-[2-[[(Benzyloxy)carbonyl]amino]ethyl]oxazole-4-carboxylate* (**23**). Cbz-β-alanine (6.41 g, 28.7 mmol) was dissolved in anhydrous CH_2_Cl_2_ (20 mL) and cooled to 0 °C in an ice bath. It was then treated with oxalyl chloride (5 mL) and stirred at 0 °C for 30 min. The reaction was next warmed to room temperature and stirred for 2.5 h. Removal of solvents *in vacuo* gave the acid chloride as a colorless oil. This was dissolved in anhydrous DMF (15 mL) and added to a solution of ethyl isocyanoacetate (2.4 mL, 22.1 mmol) and DBU (5 mL, 33.2 mmol) in anhydrous DMF (15 mL) under argon. The dark brown solution was heated to 80 °C for 4.5 h and was then poured into saturated NaHCO_3_. This was extracted with EtOAc and the combined organic layers were washed with 5% HCl, and brine. After concentration, the resulting brown oil was flash chromatographed on SiO_2_ with 15%–40% EtOAc in hexane. Oxazole **23** was isolated as a pale orange oil; 3.01 g, 43%; ^1^H-NMR δ 7.75 (s, 1H), 7.32 (m, 5H), 5.11 (m, 3H), 4.36 (q, 2H, *J =* 8), 3.55 (m, 2H), 3.28 (t, 2H, *J =* 8), 1.37 (t, 3H, *J =* 8); ^13^C-NMR δ 162.0, 157.0, 156.2, 149.4, 136.5, 128.7, 128.5, 128.4, 128.1, 128.0, 66.7, 61.2, 39.2, 26.6, 14.1; IR 3338, 3131, 3065, 3033, 2982, 2942, 2248, 1716, 1612, 1525, 1455, 1399, 1379, 1349, 1313, 1254, 1184, 1103, 1073, 1038, 912, 840, 788, 776, 736, 699, 647 cm^−1^; HRMS (ESI) *m/z* calcd for C_16_H_19_N_2_O_5_ (M+H): 318.6252; found: 318.6247.

*Ethyl 5-[2-[(tert-Butoxycarbonyl)amino]ethyl]-2-iodooxazole-4-carboxylate* (**24**). *Step A*. Compound **23** (3 g, 9.43 mmol) was dissolved in EtOAc (50 mL) and di-*tert*-butyl dicarbonate (3.09 g, 14.2 mmol) and 10% Pd/C (300 mg) were added. This was stirred under 1 atm of H_2_ overnight. The reaction mixture was filtered through Celite while washing with ethyl acetate. This was concentrated *in vacuo* to give a yellow oil which was flash chromatographed on SiO_2_ with 10%-40% EtOAc in hexane. The NHBoc product was isolated as a yellow oil; 1.94 g, 72%; ^1^H-NMR δ 7.82 (s, 1H), 4.92 (br s, 1H), 4.40 (q, 2H, *J =* 8), 3.48 (m, 2H), 3.27 (t, 2H, *J =* 8), 1.41 (t, 3H, *J =* 8); ^13^C-NMR δ 162.0, 157.3, 155.8, 149.4, 128.3, 79.4, 61.2, 33.7, 28.4, 26.8, 14.3; IR 3366, 3125, 2979, 2936, 2360, 1716, 1612, 1522, 1455, 1393, 1380, 1367, 1349, 1314, 1278, 1252, 1172, 1103, 1074, 1042, 1026, 957, 869, 841, 789, 648 cm^−1^; HRMS (ESI) *m/z* calcd for C_13_H_21_N_2_O_5_ (M+H): 284.6409; found: 284.6407. *Step B*. The product from the previous reaction (1.94 g, 6.83 mmol) was dissolved in anhydrous THF (10 mL) and placed under argon. The solution was cooled to −42 °C and treated with freshly prepared LiHMDS (19 mL, 15.03 mmol, 0.8 M in THF). The solution became dark yellow in color and was stirred for 20 min. Then a solution of ZnCl_2_ (30 mL, 15.03 mmol, 0.5 M in THF) was added and a white precipitate formed. The reaction was warmed to 0 °C for 45 min and the solution became clear. Then solid iodine (2.25 g, 8.9 mmol) was added and the reaction stirred for 1 h at room temperature. The reaction mixture was poured into saturated sodium thiosulfate solution to which 25% NH_4_OH solution had been added. This was extracted with EtOAc and the combined organic layers were dried with brine and Na_2_SO_4_. Removal of the solvent *in vacuo* gave the iodooxazole **24** as an orange oil; 2.5 g, 89%; ^1^H-NMR δ 4.77 (br s, 1H), 4.38 (q, 2H, *J =* 8), 3.46 (m, 2H), 3.26 (t, 2H, *J =* 8), 1.41 (s, 9H); ^13^C-NMR δ 162.6, 160.9, 155.6, 131.9, 99.9, 79.6, 61.4, 38.7, 28.3, 26.9, 14.3; IR 3367, 2978, 2934, 1698, 1614, 1518, 1494, 1455, 1393, 1367, 1323, 1281, 1250, 1172, 1123, 1076, 1042, 1028, 843, 785, 733 cm^−1^; HRMS (ESI) *m/z* calcd for C_13_H_20_N_2_O_5_I (M+H): 411.0411; found: 411.0412.

*Ethyl 5-[2-[(tert-Butoxycarbonyl)amino]ethyl]-2-vinyloxazole-4-carboxylate* (**25**). Oxazole **24** (2.5 g, 6.1 mmol) and tributyl(vinyl)tin (2.7 mL, 9.15 mmol) were dissolved in anhydrous dioxane (20 mL) and placed under argon. Then bis(triphenylphosphine)palladium(II) dichloride (214 mg, 0.31 mmol) was added and the reaction mixture was heated for 4 h at 105 °C. After cooling the solvent was removed *in vacuo* and the resulting brown oil was flash chromatographed on SiO_2_ with 10%–30% EtOAc in hexane. Olefin **25** was isolated as a yellow oil; 1.49 g, 79%; ^1^H-NMR δ 6.59 (dd, 1H, *J =* 12,16), 6.21 (d, 1H, *J =* 16), 5.70 (dd, 1H, *J =* 12), 4.86 (br s, 1H), 4.40 (q, 2H, *J =* 8), 3.48 (m, 2H), 3.26 (t, 2H, *J =* 8), 1.41 (s, 9H); ^13^C NMR δ 162.2, 159.3, 156.8, 155.7, 129.4, 123.2, 122.8, 79.4, 61.1, 38.3, 28.3, 26.8, 14.2; IR 3357, 2978, 2935, 1714, 1607, 1520, 1453, 1393, 1380, 1366, 1326, 1278, 1250, 1176, 1097, 1046, 983, 952, 852, 769, 732 cm^−1^; HRMS (ESI) *m/z* calcd for C_15_H_23_N_2_O_5_ (M+H): 311.1601; found: 311.1600.

*Ethyl 5-[2-[(tert-Butoxycarbonyl)amino]ethyl]-2-[[(tert-butyldimethylsilyl)oxy]-1-hydroxyethyl]oxazole-4-carboxylate* (**26**). *Step A*. AD-mix-β (17 g) and methanesulfonamide (458 mg, 4.81 mmol) were dissolved in a mixture of *t*-BuOH (150 mL) and water (150 mL) and stirred at room temperature until clear. Then a solution of **25** (1.49 g, 4.81 mmol) in *t*-BuOH (25 mL) was added. The reaction stirred at room temperature for 16 h and then additional AD-mix-β (3 g) and methanesulfonamide (458 mg, 4.81 mmol) were added and the reaction stirred at room temperature for another 24 h. Then Na_2_SO_3_ (22 g) was added and the reaction stirred for 30 minutes. It was next poured into a separatory funnel and the layers were separated. The aqueous layer was extracted with EtOAc and the combined aqueous layers were dried with Na_2_SO_4_. The solvent was removed *in vacuo* to give a pale yellow oil which was purified by flash chromatography eluting with 2%–4% MeOH in CH_2_Cl_2_. The diol was obtained as a colorless oil; 901 mg, 55%; ^1^H-NMR δ 4.84 (m, 2H), 4.38 (q, 2H, *J =* 8), 4.01 (m, 2H), 3.64 (br s, 1H), 3.47 (m, 3H), 3.21 (m, 2H), 1.37 (m, 12H); ^13^C-NMR δ 162.4, 162.2, 157.3, 156.0, 129.0, 79.9, 68.4, 65.0, 61.2, 38.7, 28.3, 27.4, 14.4; IR 3406, 2979, 1693, 1520, 1368, 1252, 1168, 1093, 1046 cm^−1^; HRMS (ESI) *m/z* calcd for C_15_H_24_N_2_O_7_Na (M+Na): 367.1476; found: 367.1476. *Step B*. The diol (900 mg, 2.62 mmol) and imidazole (356 mg, 5.23 mmol) were dissolved in anhydrous DMF (10 mL) and placed under argon. The reaction mixture was cooled to 0 °C and a solution of *tert*-butyldimethylsilyl chloride (434 mg, 2.88 mmol) in DMF (2 mL) was added dropwise. This was allowed to slowly warm to room temperature and stirred for 24 h. Additional *tert*-butyldimethylsilyl chloride (120 mg, 0.8 mmol) was added and the reaction stirred at room temperature for 6 h. This was then poured into 5% HCl and extracted with CH_2_Cl_2_. The organic extracts were dried with Na_2_SO_4_ and concentrated *in vacuo* to give a colorless oil. This was flash chromatographed on SiO_2_ with 20%–40% EtOAc in hexane and product **26** was isolated as a colorless oil; 942 mg, 79%; ^1^H-NMR δ 4.82 (m, 1H), 4.80 (br s, 1H), 4.35 (q, 2H, *J =* 8), 3.93 (m, 2H), 3.42 (m, 2H), 3.20 (t, 2H, *J =* 8), 3.12 (d, 1H, *J =* 4), 1.35 (m, 12H), 0.82 (s, 9H), 0.01 (d, 6H, *J =* 4); ^13^C-NMR δ 162.1, 161.2, 157.4, 155.7, 128.7, 79.5, 68.5, 65.3, 61.2, 38.8, 28.4, 25.8, 18.2, 14.2, −5.43; IR 3365, 2955, 2931, 2858, 1717, 1614, 1518, 1463, 1392, 1367, 1326, 1252, 1176, 1127, 1096, 1046, 839, 780 cm^−1^; HRMS (ESI) *m/z* calcd for C_21_H_38_N_2_O_7_SiNa (M+Na): 481.2340; found: 481.2336.

*Ethyl 5-[2-[(tert-Butoxycarbonyl)amino]ethyl]-2-[[(tert-butyldimethylsilyl)oxy]-1-(methanesulfonyl­oxy)ethyl]oxazole-4-carboxylate* (**27**). Prepared using the procedure detailed above for **18**, *Step B*. Concentration *in vacuo* afforded product **27** as a colorless oil; 958 mg, 97%; ^1^H-NMR δ 5.58 (dd, 1H, *J* = 4,8), 4.79 (br s, 1H), 4.32 (q, 2H, *J* = 8), 4.08 (m, 2H), 3.38 (m, 2H), 3.17 (m, 2H), 3.02 (m, 4H), 1.32 (m, 12H), 0.80 (s, 9H), 0.01 (d, 6H, *J* = 4); ^13^C-NMR δ 161.7, 158.3, 156.7, 155.7, 129.2, 79.5, 75.2, 63.3, 61.4, 45.9, 38.8, 28.3, 27.1, 25.7, 18.2, 14.3, −5.38; IR 3407, 2933, 2858, 2251, 1716, 1611, 1513, 1473, 1366, 1253, 1175, 1133, 1094, 1031, 971, 918, 839, 782, 735, 668, 647 cm^−1^; HRMS (ESI) m/z calcd for C_22_H_41_N_2_O_6_SiS (M+H): 537.2297; found: 537.2281.

*Ethyl 2-[1-Amino-2-[(tert-butyldimethylsilyl)oxy]ethyl]-5-[2-[(tert-butoxycarbonyl)amino]ethyl]oxazole-4-carboxylate* (**28**). *Step A*. Prepared using the procedure detailed above for **13**. Concentration *in vacuo* afforded the azide as a colorless oil; 813 mg, 94%; ^1^H-NMR δ 4.76 (br s, 1H), 4.57 (m, 1H), 4.30 (q, 2H, *J =* 8), 4.01 (m, 2H), 3.38 (m, 2H), 3.16 (t, 2H, *J =* 8), 1.31 (m, 12H), 0.80 (s, 9H), 0.00 (d, 6H, *J =* 4); ^13^C-NMR δ 160.8, 157.1, 156.8, 154.7, 127.9, 78.4, 63.5, 60.3, 58.7, 37.7, 27.3, 25.9, 24.6, 17.1, 13.3, −6.53; IR 3372, 2932, 2858, 2107, 1716, 1613, 1514, 1464, 1366, 1323, 1253, 1175, 1096, 1031, 839, 780, 733 cm^−1^; HRMS (ESI) *m/z* calcd for C_21_H_38_N_5_O_6_Si (M+H): 484.2586; found: 484.2578. *Step B.* Prepared using the procedure detailed above for **4a**, *Step B*. Product **28** was obtained as a pale yellow oil; 510 mg, 66%; ^1^H-NMR δ 4.78 (br s, 1H), 4.36 (q, 2H, *J =* 8), 4.13 (m, 1H), 3.90 (m, 2H), 3.42 (m, 2H), 3.18 (t, 2H, *J =* 8), 2.29 (br s, 2H), 1.36 (m, 12H), 0.82 (s, 9H), −0.01 (d, 6H, *J =* 4); ^13^C-NMR δ 162.3, 157.0, 156.5, 155.7, 128.6, 79.4, 65.9, 61.2, 52.1, 39.9, 28.4, 26.8, 25.8, 18.2, 14.4, −5.44; IR 3379, 2931, 2857, 1716, 1615, 1518, 1463, 1366, 1252, 1174, 1095, 838, 779 cm^−1^; HRMS (ESI) *m/z* calcd for C_21_H_40_N_3_O_6_Si (M+H): 458.2681; found: 458.2671.

*Ethyl 5-[2-[(tert-butoxycarbonyl)amino]ethyl]-2-[2-[(tert-Butyldimethylsilyl)oxy]-1-[[6-[[1-(4-methoxycarbonyl)oxazol-2-yl]-2-[(triiso-propylsilyl)oxy]ethyl]carbamoyl]picolinamido]ethyl]oxazole-4-carboxylate* (**30**). *Step A*. 2,6-Pyridinedicarboxylate (366 mg, 2.2 mmol) and oxazole **29** [17] (500 mg, 1.46 mmol) were dissolved in anhydrous DMF (5 mL) and placed under argon. The solution was cooled to 0 °C and treated dropwise with a solution of EDC (288 mg, 1.46 mmol), HOBt (197 mg, 1.46 mmol) and 2,6-lutidine (0.34 mL, 2.92 mmol) in DMF (5 mL). The reaction was kept at low temperature for 4 h and then warmed to room temperature and stirred overnight. This was then poured into brine and extracted with EtOAc. The combined organic extracts were washed sequentially with 10% sodium bicarbonate, 5% HCl, water and brine. This was dried with Na_2_SO_4_ and the solvent was removed under reduced pressure to give a colorless oil. The residue was flash chromatographed on SiO_2_ eluting with 15%–50% EtOAc/hexane to give 196 mg of the amide as a colorless oil, 27%; ^1^H-NMR δ 9.28 (d, 1H, *J* = 12), 8.37 (d, 1H, *J* = 8), 8.31 (d, 1H, *J* = 8), 8.15 (s, 1H), 8.00 (m, 1H), 5.60 (m, 1H), 4.20 (m, 2H), 3.80 (s, 3H), 0.88 (m, 21H); ^13^C-NMR δ 165.6, 164.1, 163.4, 161.1, 149.1, 146.1, 144.0, 139.1, 133.3, 127.5, 126.4, 64.4, 52.1, 49.7, 17.7, 11.7; IR 3316, 2945, 2867, 1748, 1685, 1584, 1525, 1456, 1345, 1252, 1203, 1114, 1072, 1000, 918, 882, 848, 801, 734, 684, 642 cm^−^^1^; HRMS (ESI) *m/z* calcd. for C_23_H_34_N_3_O_7_Si (M+H): 492.2161; found: 492.2145*. Step B.* The amide from above (196 mg, 0.4 mmol) and oxazole **28** (182 mg, 0.4 mmol) were treated as detailed above in *stepA*. Flash chromtography on SiO_2_ eluting with 10%–40% EtOAc/hexane gave the product **30** as a colorless oil, 245 mg, 66%; ^1^H-NMR δ 8.37 (d, 1H, *J* = 4), 8.35 (m, 3H), 8.25 (s, 1H), 8.21 (s, 1H), 8.01 (m, 1H), 5.60 (m, 1H), 5.47 (m, 1H), 4.84 (m, 1H), 4.35 (q, 2H, *J* = 8), 4.25 (m, 2H), 4.13 (m, 2H), 3.81 (s, 3H), 3.31 (m, 2H), 3.15 (m, 2H), 1.22 (m, 12H), 0.99 (m, 21H), 0.78 (s, 9H), −0.01 (m, 6H); ^13^C-NMR δ 171.1, 163.6, 163.4, 163.0, 162.9, 161.9, 161.3, 160.4, 157.2, 155.8, 148.5, 144.2, 138.9, 133.4, 129.0, 125.7, 79.3, 64.2, 63.6, 60.3, 52.1, 50.2, 49.9, 38.7, 28.2, 25.6, 21.0, 17.8, 14.2, 11.8, −5.50; IR 3337, 2945, 2866, 1719, 1683, 1583, 1524, 1464, 1444, 1366, 1324, 1252, 1172, 1115, 1000, 919, 882, 840, 780, 733, 646 cm^−^^1^; HRMS (ESI) *m/z* calcd for C_44_H_70_N_6_O_12_Si_2_Na (M+Na): 953.4482; found: 953.4459.

*Diethyl 2,2'-[[(Pyridine-2,6-dicarbonyl)bis(azanediyl)]bis[2-[(triisopropylsilyl)oxy]ethane-1,1-diyl]]­bis[5-[2-[(tert-butoxycarbonyl)amino]ethyl]oxazole-4-carboxylate]* (**31**). 2,6-Pyridinedicarboxylic acid (37 mg, 0.22 mmol), amine **28** (200 mg, 0.44 mmol) were treated as detailed above for **30**, *Step A.* Product **31** was obtained as a colorless oil; 195 mg, 85%; ^1^H-NMR δ 8.36 (m, 2H), 8.02 (m, 1H), 5.52 (m, 1H), 4.36 (m, 4H), 4.13 (m, 2H), 3.42 (m, 2H), 3.17 (m, 2H), 1.30 (m, 24H), 0.80 (s, 18H), 0.00 (s, 12H); ^13^C-NMR δ 163.2, 160.8, 157.1, 156.0, 148.6, 138.9, 127.1, 125.7, 79.4, 63.6, 61.0, 49.8, 38.7, 28.3, 26.9, 25.6, 21.3, 18.0, -5.2; IR 3343, 2931, 2858, 1716, 1615, 1525, 1463, 1392, 1367, 1348, 1325, 1253, 1175, 1122, 1094, 1032, 1003, 919, 840, 780, 735 cm^−1^; HRMS (ESI) *m/z* calcd for C_49_H_79_N_7_O_14_Si_2_Na (M+Na): 1068.5116; found: 1068.5117.

*5-[2-[(tert-Butoxycarbonyl)amino]ethyl]-2'-[6-[4-carboxy-[2,4'-bioxazol]-2'-yl]pyridine-2-yl]-[2,4'-bioxazole]-4-carboxylic acid* (**32**). *Step A*. **30** (245 mg, 0.26 mmol) was dissolved in anhydrous THF (10 mL) and pyridine (1 mL) and HF-pyridine complex (0.3 mL) was added. The reaction was stirred at room temperature overnight and was then poured into saturated sodium bicarbonate solution. This was extracted with CH_2_Cl_2_ and dried with Na_2_SO_4_. Removal of solvent under vacuum gave 174 mg of the diol as a colorless oil, 100%; ^1^H-NMR δ 8.17 (m, 4H), 7.78 (m, 1H), 5.58 (m, 2H), 5.12 (s, 1H), 4.27 (m, 6H), 3.83 (s, 3H), 3.44 (m, 2H), 3.21 (t, 2H, *J* = 8), 1.36 (s, 9H), 1.08 (t, 3H, *J* = 8); ^13^C-NMR δ 161.2, 161.7, 161.4, 160.9, 160.2, 158.6, 157.7, 155.9, 149.5, 148.1, 144.4, 138.5, 136.2, 132.8, 128.1, 125.3, 79.4, 70.3, 62.0, 52.1, 38.4, 29.2, 28.3, 25.0, 22.6, 17.1; IR 3333 (br), 2954, 2250, 1720, 1678, 1617, 1582, 1530, 1442, 1367, 1347, 1324, 1275, 2248, 1174, 1114, 1000, 963, 915, 846, 805, 774, 734, 705, 646 cm^−^^1^; HRMS (ESI) *m/z* calcd for C_29_H_36_N_6_O_12_Na (M+Na): 683.2283; found: 683.2262. *Step B*. The diol (174 mg, 0.26 mmol) was dissolved in anhydrous CH_2_Cl_2_ (7 mL) and placed under argon. The flask was cooled to −78 °C and the solution was treated with DAST (87 μL, 0.66 mmol). The reaction stirred at low temperature for 4 h and then solid K_2_CO_3_ (81 mg, 0.66 mmol) was added. The reaction was warmed to room temperature and poured into saturated sodium bicarbonate solution. This was extracted with CH_2_Cl_2_ and the combined organic extracts were dried with Na_2_SO_4_. The solvent was removed *in vacuo* to give the bis(oxazoline) 153 mg as a yellow oil, 93%; ^1^H-NMR δ 8.26 (m, 3H), 7.94 (t, 1H, *J* = 8), 5.63 (m, 2H), 4.92 (m, 5H), 4.38 (m, 2H), 3.92 (s, 3H), 3.46 (m, 2H), 3.24 (t, 2H, *J* = 8), 1.41 (s, 9H); ^13^C-NMR δ 165.1, 164.9, 163.0, 161.9, 161.3, 160.6, 158.1, 155.7, 146.3, 146.1, 144.8, 137.6, 133.4, 128.9, 126.8, 126.7, 79.3, 71.3, 68.4, 64.0, 61.2, 52.2, 42.1, 38.7, 29.6, 28.3, 14.3; IR 3381, 2977, 2931, 1716, 1639, 1582, 1518, 1460, 1366, 1345, 1322, 1249, 1176, 1144, 1112, 1033, 978, 918, 833, 804, 734 cm^−^^1^; HRMS (ESI) m/z calcd for C_29_H_32_N_6_O_10_Na (M+Na): 647.2072; found: 647.2054. *Step C*. The bis(oxazoline) (153 mg, 0.25 mmol) was dissolved in anhydrous CH_3_CN and placed under argon. The flask was cooled to 0 °C and the solution was treated drop-wise sequentially with DBU (156 μL 1.04 mmol) and BrCCl_3_ (123 μL, 1.25 mmol). The reaction was gradually warmed to room temperature and stirred overnight. A white solid precipitated and was filtered and washed with CH_3_CN. The solid was dried to give 91 mg of the tetraoxazole diester as a white solid, 60%; mp 222 °C (dec); ^1^H-NMR δ 8.42 (s, 1H), 8.37 (s, 1H), 8.31 (m, 3H), 8.07 (t, 1H, *J* = 8), 4.85 (s, 1H), 4.42 (m, 2H), 5.31 (s, 3H), 3.56 (m, 2H), 3.35 (m, 2H), 1.42 (s, 9H); ^13^C NMR δ 162.0, 161.3, 160.8, 160.5, 157.3, 155.4, 153.3, 145.6, 145.5, 144.0, 141.1, 138.5, 134.5, 131.5, 131.3, 129.9, 124.4, 124.2 79.5, 61.4, 54.4, 38.9, 28.4, 27.0, 14.4; IR 3356, 3111, 2978, 1719, 1574, 1523, 1453, 1367, 1325, 1253, 1158, 1098, 1045, 998, 971, 926, 824, 780, 734, 712 cm^−^^1^. *Step D*. The diester (71 mg, 0.13 mmol) was suspended in a mixture of THF (30 mL) and water (3 mL) and lithium hydroxide (12 mg, 0.29 mmol) was added. The reaction was refluxed for 30 min and then stirred at room temperature overnight. THF was removed under vacuum and 5% HCl was added to the remaining solution. A white solid precipitated and was filtered and washed with water. The solid was dried by azeotroping with toluene 3 times to give 50 mg of diacid **32**, as a white solid, 67%; mp 225–226 °C; HRMS (ESI) m/z calcd for C_26_H_22_N_6_O_10_Na (M+Na): 601.1290; found: 601.1284.

*2,2'-(Pyridine-2,6-diyl)bis[5-[(tert-butoxycarbonyl)amino]ethyl]-[2,4'-bioxazole]-4-carboxylic acid* (**33**). *Step A*. Prepared using the procedure detailed above for **32** *Step A*. The diol was obtained as a yellow oil, 134 mg, 89%; ^1^H-NMR δ 8.60 (s, 2H), 7.67 (m, 1H), 5.50 (m, 1H), 4.23 (m, 4H), 3.45 (m, 2H), 3.15 (m, 2H), 1.36 (m, 24H); ^13^C-NMR δ 163.6, 161.9, 157.8, 156.0, 155.9, 148.5, 125.3, 123.8, 79.5, 62.9, 61.2, 53.5, 37.7, 28.3, 27.1, 14.2; IR 3339, 2978, 2934, 2248, 1716, 1616, 1529, 1445, 1367, 1347, 1325, 1281, 1250, 1174, 1092, 1047, 1000, 917, 846, 788, 733, 706 cm^−1^; HRMS (ESI) *m/z* calcd for C_37_H_51_N_7_O_14_Na (M+Na): 840.3386; found: 840.3383. *Step B*. Prepared using the procedure detailed above for **32** *Step B*. The bis(oxazoline) was obtained as an orange oil; 116 mg, 91%; ^1^H-NMR δ 8.25 (d, 2H, *J =* 8), 7.93 (t, 1H, *J =* 8), 5.61 (t, 2H, *J =* 8), 4.91 (m, 6H), 4.39 (q, 4H, *J =* 8), 3.46 (m, 4H), 3.24 (t, 4H, *J =* 8), 1.41 (m, 24H); ^13^C-NMR δ 164.9, 162.0, 160.6, 158.1, 155.7, 146.2, 137.6, 128.9, 126.8, 79.5, 71.3, 64.0, 61.2, 38.7, 28.3, 18.9; IR 3364, 2977, 1713, 1520, 1458, 1366, 1250, 1175, 1093, 1031, 921, 844, 733 cm^−1^; HRMS (ESI) *m/z* calcd for C_37_H_47_N_7_O_12_Na (M+Na): 804.3175; found: 804.3167. *Step C*. Prepared using the procedure detailed above for **32 ***Step C*. This was purified by flash chromatography eluting with 1%–4% MeOH/CH_2_Cl_2_. The tetra-oxazole diester was isolated as a white solid; 58 mg, 50%; mp 245–247 °C; ^1^H-NMR (CDCl_3_ + CD_3_OD) δ 8.55 (s, 2H), 8.43 (d, 2H, *J =* 4), 8.08 (t, 1H, *J =* 8), 5.13 (br s, 2H), 4.44 (d, 4H, *J =* 8), 3.56 (m, 4H), 3.36 (m, 4H), 1.42 (m, 24H); ^13^C-NMR (CDCl_3_ + CD_3_OD) δ 161.8, 160.4, 157.3, 155.7, 153.2, 145.5, 140.6, 138.4, 131.4, 129.7, 124.2, 79.3, 61.3, 38.1, 28.3, 27.0, 14.3; IR 3355, 2978, 1710, 1639, 1524, 1452, 1367, 1250, 1171, 1089, 1048, 926, 733 cm^−1^; HRMS (ESI) *m/z* calcd for C_37_H_43_N_7_O_12_Na (M+Na): 800.2862; found: 800.2853*. Step D*. Prepared using the procedure detailed above for **32** *Step D*. Diacid **33** was obtained as a white solid; 35 mg, 65%; mp 250–252 °C (dec.); ^1^H-NMR (CDCl_3_ + CD_3_OD) δ 8.28 (s, 2H), 8.11 (d, 2H, *J =* 4), 7.82 (t, 1H, *J =* 8), 5.44 (br s, 2H), 3.22 (m, 4H), 3.05 (m, 4H), 1.13 (s, 18H); ^13^C-NMR (CDCl_3_ + CD_3_OD) δ 168.2, 165.1, 161.9, 157.8, 157.7, 150.3, 145.3, 143.3, 136.4, 134.8, 128.9, 83.6, 43.6, 35.1, 19.1; IR 3439, 2977, 2253, 2127, 1702, 1525, 1453, 1392, 1366, 1342, 1281, 1250, 1173, 1026, 926, 824, 762, 710 cm^−1^; HRMS (ESI) *m/z* calcd for C_33_H_35_N_7_O_12_Na (M+Na): 744.2236; found: 744.2229.

*Pyridyl tetraoxazole macrocycle with a single 2-[[(tert-butoxy)carbonyl]amino]ethyl side chain* (**34**). Prepared from **32** using the procedure detailed above for **8**. Flash chromatography on SiO_2_ eluting with 1%–6% MeOH/CH_2_Cl_2_ gave macrocycle **34** as a white solid, 8 mg, 20%; mp 144–145 °C; ^1^H-NMR δ 8.45 (m, 3H), 8.27 (m, 3H), 8.08 (m, 2H), 7.49 (m, 5H), 5.29 (s, 1H), 4.58 (m, 4H), 3.55 (m, 2H), 3.37 (t, 2H, *J =* 8), 1.43 (s, 9H); ^13^C-NMR δ 161.0, 160.5, 160.3, 159.7, 156.1, 154.3, 145.6, 145.5, 140.5, 139.0, 138.4, 138.5, 137.7, 123.5, 131.9, 130.9, 129.9, 129.6, 129.5, 128.8, 122.6, 79.5, 43.8, 39.2, 29.0, 28.4; IR 3390, 2959, 2929, 2859, 1727, 1666, 1594, 1516, 1463, 1366, 1274, 1170, 1122, 1073, 991, 777, 738, 709 cm^−1^; HRMS (ESI) *m/z* calcd for C_34_H_30_N_8_O_8_Na (M+Na): 701.2079; found: 701.2069.

*Pyridyl tetraoxazole macrocycle with two 2-[[(tert-butoxy)carbonyl]amino]ethyl side chains* (**35**). Prepared from **33** using the procedure detailed above for **8**. Flash chromatography eluting with 1–5% MeOH/CH_2_Cl_2_ to give macrocycle **35** as a white solid; 11 mg, 28%; mp 170–173 °C; ^1^H-NMR δ 8.24 (s, 2H), 8.06 (d, 2H, *J =* 8), 7.58 (s, 1H), 7.37 (m, 4H), 5.26 (s, 2H), 4.57 (d, 4H, *J =* 4), 3.55 (m, 4H), 3.37 (t, 4H, *J =* 8), 1.43 (s, 18H); ^13^C-NMR δ 161.1, 160.4, 156.1, 153.7, 152.2, 145.5, 138.8, 138.4, 137.9, 131.9, 131.5, 129.8, 129.4, 122.7, 79.3, 43.8, 39.2, 28.4, 26.4; IR 3322, 2976, 1695, 1646, 1525, 1440, 1366, 1284, 1250, 1170, 1093, 1047, 992, 926, 780, 734, 707 cm^−1^; HRMS (ESI) *m/z* calcd for C_41_H_43_N_9_O_10_Na (M+Na): 844.3025; found: 844.3024.

*Pyridyl tetraoxazole macrocycle with a single 2-(N,N-dimethylamino)ethyl side chain* (**36**). *Step A*. Prepared from **34** using the procedure detailed above for **22** *Step A*. The crude product was washed with hexane and dried to give 6 mg of the salt as a white solid, 100%; mp 170 °C (dec); ^1^H-NMR (DMSO-d_6_) δ 9.17 (s, 1H), 9.06 (s, 1H), 8.85 (s, 1H), 8.26 (m, 4H), 7.93 (m, 3H), 7.36 (m, 3H), 4.49 (m, 4H), 3.46 (m, 2H), 3.25 (m, 2H); IR 2958, 2918, 2855, 2351, 1726, 1671, 1650, 1595, 1540, 1507, 1441, 1266, 1178, 1111, 986, 915, 833, 800, 707 cm^−^^1^; HRMS (free-base form) (ESI) *m/z* calcd for C_29_H_22_N_8_O_6_Na (M+H): 579.1735; found: 579.1758. *Step B*. Prepared using the procedure detailed above for **11 ***Step B*. Product **36** was obtained as a white solid; 4 mg, 100%; mp 150–155 °C; ^1^H NMR (CDCl_3_ + CD_3_OD) δ 8.36 (s, 1H), 8.35 (s, 1H), 8.33 (s, 1H), 8.11 (m, 3H), 7.37 (m, 4H), 4.60 (m, 4H), 3.40 (m, 2H), 2.46 (s, 6H); ^13^C-NMR (CDCl_3_ + CD_3_OD) δ 161.0, 141.5, 139.3, 137.8, 129.5, 129.2, 129.1, 123.0, 122.9, 57.0, 44.9, 43.6, 23.9; IR 3395, 2964, 2918, 2849, 2351, 1732, 1661, 1655, 1595, 1545, 1514, 1463, 1447, 1370, 1321, 1266, 1173, 1108, 992, 926, 921, 822, 734, 722 cm^−^^1^; HRMS (ESI) *m/z* calcd for C_31_H_26_N_8_O_6_ 607.2048; found: 607.2069.

*Pyridyl tetraoxazole macrocycle with two 2-(N,N-dimethylamino)ethyl side chain*s (**37**). *Step A.* Prepared from **35** using the procedure detailed above for **22** *Step A*. The bis(trifluoroacetate) salt was obtained as a white solid; 11 mg, 100%; mp 260 °C (dec.); ^1^H-NMR (CD_3_OD) δ 8.63 (s, 2H), 8.57 (s, 4H), 7.53 (s, 1H), 7.30 (s, 4H), 4.48 (s, 4H), 3.42 (s, 4H); IR 3405, 2964, 2926, 2855, 1653, 1529, 1452, 1266, 1025, 992, 827, 800 cm^−1^; HRMS (ESI) *m/z* calcd for C_31_H_28_N_9_O_6_ (M+H): 622.2142; found: 622.2140. *Step B*. Prepared using the procedure detailed above for **11** *Step B*. Bis(dimethylamino) product **37** was obtained as a white solid; 6 mg, 100%; mp 180–184 °C; ^1^H-NMR (CDCl_3_ + CD_3_OD) δ 8.31 (s, 2H), 8.08 (s, 3H), 7.52 (s, 1H), 7.35 (s, 3H), 5.32 (s, 4H), 2.80 (t, 4H, *J =* 8), 2.39 (m, 16H); ^13^C-NMR (CDCl_3_ + CD_3_OD) δ 162.5, 160.9, 145.4, 139.0, 129.4, 129.1, 122.8, 64.5, 44.9, 43.7, 23.9; HRMS (ESI) *m/z* calcd for C_35_H_36_N_9_O_6_ (M+H): 678.2789; found: 678.2793.

*Temperature-Dependent Spectrophotometry***.** Temperature-dependent absorption experiments were conducted on an AVIV Model 14DS Spectrophotometer (Aviv Biomedical, Lakewood, NJ, USA) equipped with a thermoelectrically controlled cell holder. Quartz cells with a path length of 1.0 cm were used for all the absorbance studies. Temperature-dependent absorption profiles were acquired at either 260 nm (for ST duplex DNA) or 295 nm (for hTel quadruplex DNA) with a 5 s averaging time. The temperature was raised in 0.5 °C increments, and the samples were allowed to equilibrate for 1 min at each temperature setting. In the quadruplex melting studies, the hTel concentration was 5 µM in strand (120 μM in nucleotide). When present in these quadruplex studies, the drug concentrations were 20 µM. In the duplex melting studies the ST DNA concentrations were 15 µM base pair (30 μM in nucleotide) and, when present, the drug concentrations were 15 µM. The buffer for all the UV melting experiments contained 10 mM potassium phosphate (pH 7.5) and sufficient KCl (132 mM) to bring the total K^+^ concentration to 150 mM. Prior to their use in the UV melting experiments, all nucleic acid solutions were preheated at 90 °C for 5 min and slowly cooled to room temperature over a period of 4 hr.

### 3.2. Cytotoxicity Assays

Cytotoxicity was determined using the MTT-microtiter plate tetrazolinium assay (MTA). The human lymphoblast RPMI 8402 cell line was provided by Dr. Toshiwo Andoh (Aichi Cancer Center Research Institute, Nagoya, Japan) [38]. The KB3-1 cell line was obtained from K.V. Chin (The Cancer Institute of New Jersey, New Brunswick, NJ, USA) [39]. The cytotoxicity assay was performed using 96-well microtiter plates. Cells were grown in suspension at 37 °C in 5% CO_2_ and maintained by regular passage in RPMI medium supplemented with 10% heat inactivated fetal bovine serum, l-glutamine (2 mM), penicillin (100 U/mL), and Streptomycin (0.1 mg/mL). For determination of IC_50_ values, cells were exposed continuously for four days to varying concentrations of drug, and MTT assays were performed at the end of the fourth day. Each assay was performed with a control that did not contain any drug. All assays were performed at least twice in six replicate wells.

## 4. Conclusions

The results from this structure-activity investigation of macrocyclic pyridyl polyoxazoles indicate that analogs that have either a dimethylamino group directly attached to, or separated from the phenyl ring at the 4- or 5-positions by two methylene groups strongly and selectively stabilize G-quadruplex DNA. These same analogs are also highly cytotoxic against KB3-1 cells with IC_50_ values ≤ 70 nM. A dimethylaminomethyl group at the 5-position of the phenyl is essentially devoid of G-quadruplex stabilizing and cytotoxic activity. Extending the side-chain by one methylene group to form a propyl chain at either the 4- or 5-position of the phenyl ring fails to improve cytotoxic activity over the corresponding ethyl analogs, although the 5-substituted analog does strongly stabilize G-quadruplex DNA. Attaching a 2-(dimethylamino)ethyl chain to an oxazole instead of the phenyl ring results in an analog with moderate cytotoxic activity but low G-quadruplex stabilizing capability. It is conceivable that this compound might have affinity for other types of G-quadruplexes, perhaps RNA, which might account for its modest cytotoxic activity. Upon attaching a second such side chain onto another oxazole ring quadruplex stabilization and cytotoxic activity are both diminshed. These studies suggest that when selective G-quadruplex stabilization, cytotoxic activity, water-solubility, and ease of synthesis are all taken into account, the previously-reported 5-[2-(dimethylamino)ethyl]phenyl analog **2** represents one of the better compounds for further development.
